# Quantification of the growth suppression of HER2+ breast cancer colonies under the effect of trastuzumab and PD-1/PD-L1 inhibitor

**DOI:** 10.3389/fonc.2022.977664

**Published:** 2022-12-21

**Authors:** Regina Padmanabhan, Hadeel Kheraldine, Ishita Gupta, Nader Meskin, Anas Hamad, Semir Vranic, Ala-Eddin Al Moustafa

**Affiliations:** ^1^ Department of Electrical Engineering, Qatar University, Doha, Qatar; ^2^ College of Medicine, Qatar University (QU) Health, Qatar University, Doha, Qatar; ^3^ Biomedical Research Centre, Qatar University, Doha, Qatar; ^4^ Pharmaceutical Department at Hamad Medical Corporation, Hamad Medical Corporation, Doha, Qatar

**Keywords:** HER2, PD-1/PD-L1, mathematical model, HER2/PD-1 interaction, breast cancer

## Abstract

**Introduction:**

Immune checkpoint blockade (ICB)-based therapy is revolutionizing cancer treatment by fostering successful immune surveillance and effector cell responses against various types of cancers. However, patients with HER2+ cancers are yet to benefit from this therapeutic strategy. Precisely, several questions regarding the right combination of drugs, drug modality, and effective dose recommendations pertaining to the use of ICB-based therapy for HER2+ patients remain unanswered.

**Methods:**

In this study, we use a mathematical modeling-based approach to quantify the growth inhibition of HER2+ breast cancer (BC) cell colonies (ZR75) when treated with anti-HER2; trastuzumab (TZ) and anti-PD-1/PD-L1 (BMS-202) agents.

**Results and discussion:**

Our data show that a combination therapy of TZ and BMS-202 can significantly reduce the viability of ZR75 cells and trigger several morphological changes. The combination decreased the cell’s invasiveness along with altering several key pathways, such as Akt/mTor and ErbB2 compared to monotherapy. In addition, BMS-202 causes dose-dependent growth inhibition of HER2+ BC cell colonies alone, while this effect is significantly improved when used in combination with TZ. Based on the in-vitro monoculture experiments conducted, we argue that BMS-202 can cause tumor growth suppression not only by mediating immune response but also by interfering with the growth signaling pathways of HER2+BC. Nevertheless, further studies are imperative to substantiate this argument and to uncover the potential crosstalk between PD-1/PD-L1 inhibitors and HER2 growth signaling pathways in breast cancer.

## Introduction

Recently, the inevitable role of executable, integrated, mathematical, and computational models in cancer research was largely acknowledged and discussed in many recent reviews (1–4). It is apparent that an integrated approach, which involves the analysis of genomic profiles, histopathology, imaging data, immunohistochemistry, proteomics data, drug targets, drug response, and more are imperative to coin translational solutions for cancer management. Specifically, the important role of mathematical and computational models in (1): illustrating highly dynamic biological behaviors (2), quantifying disease characteristics and drug responses (3), allowing easy integration of structured control-theoretic methods for the design of appropriate intervention strategies, and (4) utilizing intelligent algorithms to facilitate reasoning and decision support; are intensively explored recently (2).

HER2^+^ BC that constitutes 15-20% of all BC types is identified by the overexpression of the HER2 receptor due to *HER2/ERBB2* gene amplification (5, 6). This molecular subtype of BC is associated with poor prognosis, moreover, 30% of patients report metastasis, especially to the brain (2, 7, 8). HER2 targeted therapies have significantly improved post-treatment disease-free survival (DFS) of HER2^+^ BC patients (9, 10). However, patients undergoing current standard of care treatment (a combination of chemotherapy and anti-HER2 agents) who are under longtime follow-ups report unsatisfactory response rate (20-50%), development of drug resistance, and disease recurrence (9–12). For instance, under TZ therapy, compared to the 3 years (DFS=87.1%) follow-up, a drop of 13.4% in DFS was reported in the case of 10 years (DFS=73.7%) follow-up (13). Similarly, a drop in DFS was reported with a treatment strategy that used a combination of pertuzumab, trastuzumab, docetaxel, and trastuzumab emtansine (14, 15). Hence, there is a quest for the development of computationally and experimentally driven therapeutic strategies for the better management of HER2^+^ BC patients.

Modern immunotherapeutic strategies which include the use of ICBs are increasingly recommended for the treatment of many types of cancers (16). The fact that scientists behind the identification of programmed death (PD-1) protein were honored with the Nobel prize (2018) signifies the potential benefits of this discovery in cancer therapy. In line with what was expected, several experimental and clinical trials substantiated the credibility of ICBs in terms of (1): safety, potency, and commercial availability (2), memory-lymphocyte mediated long term immunity that leads to durable complete response, and (3) additional advantages in treating advanced and metastatic cancers. For instance, compared to conventional treatment, augmenting ICB-based therapy has shown improved treatment response in many cancers which were otherwise not manageable or relapsing (e.g. melanoma, non-small cell lung cancer). However, the role of ICBs in BC treatment is in its emerging stage. Two important milestones in this regard are the approval of monoclonal antibodies (mAbs) atezolizumab (anti-PD-L1, March 2019) and pembrolizumab (anti-PD-1, November 2020) for the treatment of triple-negative BC (TNBC) (17–21).

Similar to TNBC, the disease progression in HER2^+^ BC patients have shown a considerable correlation with the immune response and hence it is hypothesized that ICB-based immunomodulation techniques can be used in a favorable way to manage this aggressive cancer as well (19, 20). Many clinical and preclinical experiments associate poor disease prognosis in the case of HER2^+^ BC with the expression of PD-L1 which might have aided this type of cancers to hide from immune surveillance (19, 20, 22–25). Moreover, studies report increased expression of PD-L1 under treatment with TZ (26). With one of the rationales identified behind the refractory nature of HER2^+^ BC after anti-HER2 treatment as upregulation of immune checkpoints such as PD-1/PD-L1 and CTLA-4, amending ICB-based treatment is thought to add therapeutic benefits in treating HER2^+^ BC (22, 27, 28). In line with these indications, reviews suggested that patients with metastatic breast cancer should be tested for response to ICBs for better treatment options (29). Consequently, several ICB-based agents are currently under investigation for the management of HER2^+^ BC, however, none of them have been approved yet (2, 7). ICB-based drugs being a novel investigational therapeutic option for HER2^+^ BC, it is imperative to come up with a quantitative comparison against current standard treatment options (4).

Preliminary investigations towards the advantages of combining anti-HER2 treatment with ICB-based therapy also suggest modest and durable outcome in a proportion of HER2^+^ patients, which is another promising lead that calls for more investigations in this area (25, 30, 31). Apart from mAbs, other drug modalities including small molecules, peptides, and macrocycles are also available for inducing ICB-based therapy (32). Due to the reported resistance to mAb-based therapy and relapse after treatment, there is an increased interest in other drug modalities as well (33–35). Some of the disadvantages of mAbs are difficulty in production, longer half-life, high molecular weight, and less diffusion, on the other hand, small molecules have good affinity, oral bioavailability, and lesser immunotoxicity compared with mAbs (34, 36). Tight binding and retention of mAbs often leads to increased immune-related adverse events (irAEs) compared to small molecule inhibitors (SmIs) (37). Thus, SmIs that block interaction between PD-1 receptor and PD-L1 (ligand) are considered as a promising alternative to many of the currently investigated mAbs. Consequently, there is an apparent need for more research on the development and use of anti-PD-1/PD-L1 SmIs.

Mathematical modeling allows the integration of observed (empirical) results pertaining to a complex biological phenomenon in a simplified way and enables theoretical analysis and simulation studies. Such models can be used for the prediction of future behavior and to study the influence of each parameter on the overall cancer dynamics. Hence, in this study, we use a mathematical modeling-based approach to develop a new model and quantify the growth inhibition of HER2^+^ BC cell colonies (ZR75) when treated with anti-HER2 (TZ) and anti-PD-1/PD-L1 (BMS-202) agents.

## Materials and methods

### Cell culture

The HER2^+^ cell-line (ZR75) was purchased from the American type culture collection (ATCC) (Rockville, MD, USA) and grown in complete cell culture media, RPMI-1640, (Gibco, Life technologies, Burlington, ON, Canada) augmented with 1% PenStrep antibiotic (Invitrogen, Life Technologies) and 10% fetal bovine serum (FBS; Invitrogen, Life Technologies). Cells were maintained at a temperature of 37°C with a 5% CO2 humidified atmosphere. We confirmed the presence of HER2 in this cell line in our previous study (38).

### Cell viability assay

ZR75 cells were seeded in 96-well plates (Thermo Fisher Scientific, Mississauga, ON, Canada) at a density of 8,000 cells/well. After 24 hours, media was replaced with a fresh one with or without the treatment. Cells were treated with TZ (0, 1, 5, 7, 10, 15, and 20 µg/mL), BMS-202 (0, 1, 5, 7, 10, 15, and 20 µM), or a combination of both for 48 hours. Then, media was replaced with Alamar Blue cell viability reagent (Invitrogen, Thermo Fisher Scientific) and cells were incubated with the dye for 4 hours in the dark at 37°C as per the manufacturer protocol. Fluorescence values were recorded at a wavelength of 560 nm (excitation) and 600 nm (emission) using the Infinite m200 PRO fluorescent microplate reader (TECAN, Männedorf, Switzerland), reflecting the number of viable cells in each well.

### Morphological examination

ZR75 cells were seeded in 6-well plates at a density of 200,000 cells/well. Changes in morphology of ZR75 cells were recorded after 48 hours of treatment with TZ (5 µg/mL), BMS-202 (5 µM), or a combination of both. Cells were visualized using Leica DMi1 inverted microscope (Leica Microsystems, Wetzlar, Germany). Untreated cells were used as a control.

### Cell invasion assay

ZR75 cells were cultured in the upper chamber of 24-wells BioCoat™ Matrigel^®^ Invasion Chambers (Corning, USA) with 8.0µm PET Membrane in a density of 50,000 cells/well. Cells were maintained in serum-free medium with/without treatment. The wells were placed in a base of complete medium with 10% FBS and incubated at 37°C for 48 hours. After that, non-invasive cells in the upper well were removed with a cotton swab. Invasive cells were washed, fixed with 4% formaldehyde, followed by staining with 300 ng/mL of DAPI (Abcam, Cambridge, MA, USA) for 2 minutes in the dark. Then, cells were observed using the fluorescence microscope.

### Western blotting

ZR75 cells were seeded in 100 mm petri dishes at a density of 2,000,000 cells/dish. Cells were treated with TZ, BMS-202, or a combination of both for 48 hours. Cell lysates were collected, and 30 μg of proteins were resolved on 10% polyacrylamide SDS PAGE gels and then transferred onto PVDF membranes. Membranes were probed with the following primary antibodies: anti-rabbit Akt (CST: 9272S), anti-rabbit phospho-Akt (Ser473) (CST: 4060S), anti-rabbit mTOR (CST: 2983S), anti-rabbit phospho mTOR (S2448) (Abcam: ab109268), anti-mouse ErbB2 (Abcam: ab16901), anti-rabbit phospho ErbB2 (Abcam: ab53290), and anti-rabbit vimentin (CST: 46173S). Anti-rabbit GAPDH (Cell Signaling: 8480S) was used to ensure equal loading of protein samples. Blots were incubated with ECL Western blotting substrate (Pierce Biotechnology, Rockford, IL, USA) and chemiluminescence was recorded using the iBrightTM CL1000 imaging system (Thermo Fisher Scientific, Wal-tham, MA, USA). Quantification was done using ImageJ software.

### Soft agar assay

Colony formation in soft agar was used to determine cells’ capacity to colonize in *in-vitro*. A total of 1×10^3^ cells of ZR75 were placed in RPMI medium containing 0.2% agar with/without drug(s) (treated and control cells, respectively) and plated in a 6-well plate covered with a layer of 0.4% noble agar in RPMI complete growth media (1 ml solid agar layer/well). A volume of 500 µl of media without (control) or with drug(s) were added to each well on 12^th^ and 14^th^ day of plating for ZR75 to make sure that the agar does not dry. The concentration range for BMS-202 was set to 1-20 µM, as our preliminary experiments on ZR75 colonies revealed no significant drug effect when treated with lower concentrations. Similar ranges were reported in (IC50 15 μM, in PD-L1+ SCC-3 cells and IC50 10 μM, in anti-CD3 activated Jurkat cells) (39), (0.6 nM up to 20 µM) (32), and (2.5-80 µM) (36) for various experiments based on different cell-lines. Colony formation was monitored every two days for a period of three weeks, and pictures of the colonies were taken on the 5^th^, 7^th^,9^th^, 12^th^, 14^th^, 17^th^, and 19^th^ day after seeding from various locations in each well using the inverted light microscope (Leica, Germany).

### Model parameter estimation

At least 3 or up to 7 sets (different colonies) of time-series data were collected for each of the 16 samples (15 concentration and 1 control) of ZR75 on every 2^nd^ or 3^rd^ day for up to 19 days. Each time-series data for a particular colony includes up to 7 data points (images captured on 5^th^, 7^th^, 9^th^, 12^th^, 14^th^, 17^th^, and 19^th^ day). All the images required for our study were taken using an inverted microscope (Leica microsystems, Germany) interfaced to LAS EZ software. In order to measure the time-dependent changes in the area of colonies, images were calibrated to 100 µm scale and quantified using ImageJ software. Matlab^®^ *lsqcurvefit()* algorithm was used to estimate model parameters. Mean and standard deviation of parameter estimates were calculated using data sets pertaining to different colonies treated with a particular concentration of drug or drug combination. More than 1200 images were collected for our mathematical modeling experiments alone (excluding preliminary ones) from different wells, out of which around 500 images were omitted as (1) on day one there were no colonies inside or around the marked area to track (2) some colonies inside the marked areas were dormant (3) in some cases at least 4 images (on different days) of the same colony were not captured. Hence, after the experiment, we ended up with 3 to 7 data sets each data set with 4 to 7 data points (days) for various drug concentrations and combinations. Since the growth of breast cancer cell line colonies are nonlinear, we required at least 3 or 4 images of the same colony on different days for model parameter estimation.

### Statistical analysis

Data are presented as an average of mean ± SEM (standard error of the mean). Each experiment was repeated at least three times (n=3). One-way ANOVA followed by Tukey’s *post-hoc* test was used to compare the difference between treated and untreated cells. The data were analyzed using Microsoft Excel, and differences with p< 0.05 were considered statistically significant.

## Results

We tested whether our HER2^+^ BC cell lines (ZR75) express the drug target, PD-L1. FACS analysis of cell surface proteins revealed that 14.2% of ZR75 cells express PD-L1 ligand (data not shown). Thus, we proceeded with the treatment and the following experiments.

**Figure 1:**
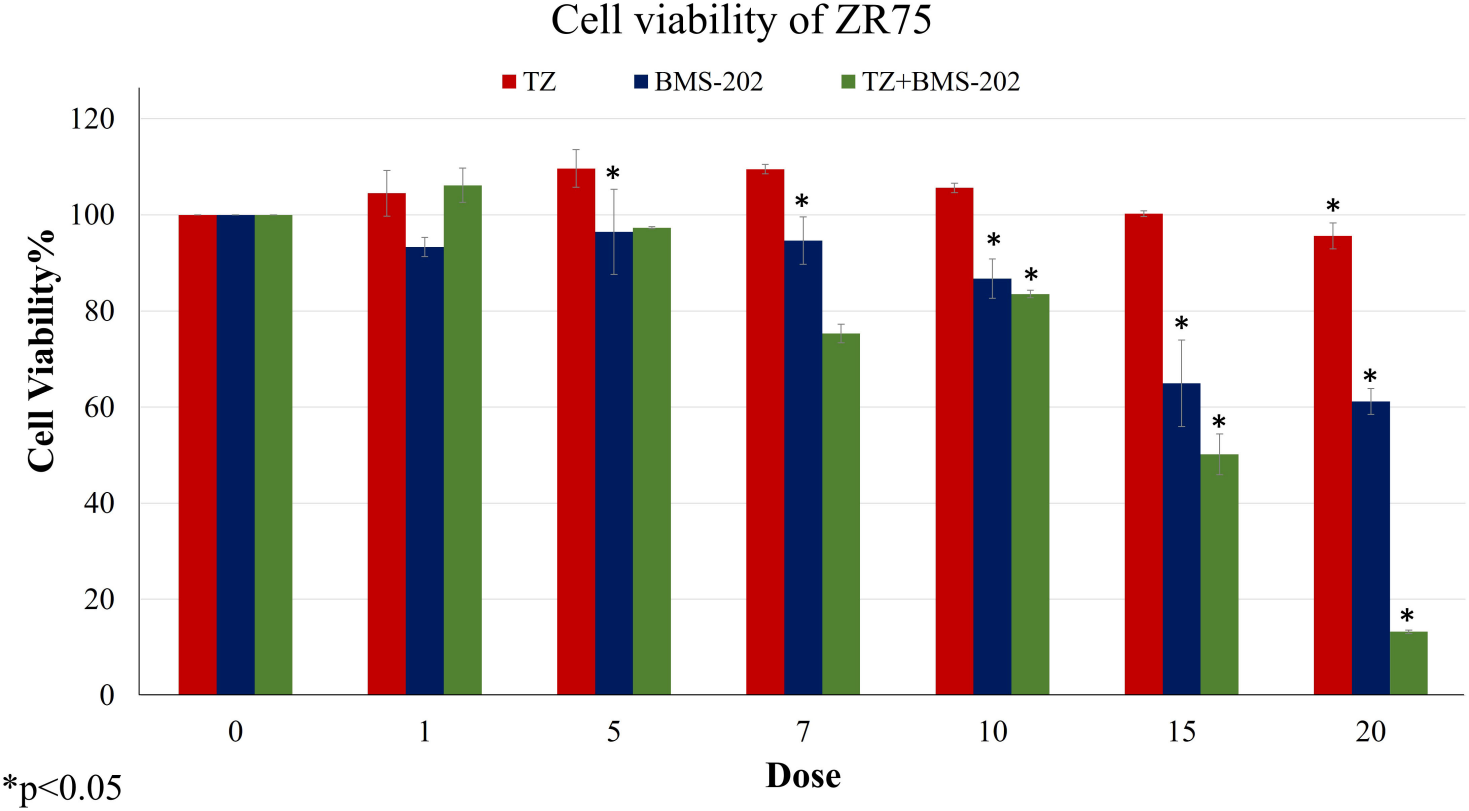
The effects of different concentrations of TZ, BMS-202, and a combination of both drugs on cell viability of ZR75 cell line. A significant dose-dependent decrease in cell viability was observed after treatment with the combination therapy. Data are presented as a percentage of viable cells ± SEM.

We first examined the outcome of TZ and BMS-202 on the viability of ZR75; a HER2^+^ BC cell line. A significant decrease in the viability of ZR75 cells was observed after mono-treatment with TZ (20µg/mL) and BMS-202 (10µM). Interestingly, combining both treatments resulted in a more significant reduction of cell viability in a dose-dependent fashion, starting from a low dose (5µg/mL of TZ + 5µM of BMS-202) and reaching 13.42 ± 0.37% at high doses (**Figure 1**).

**Figure 2:**
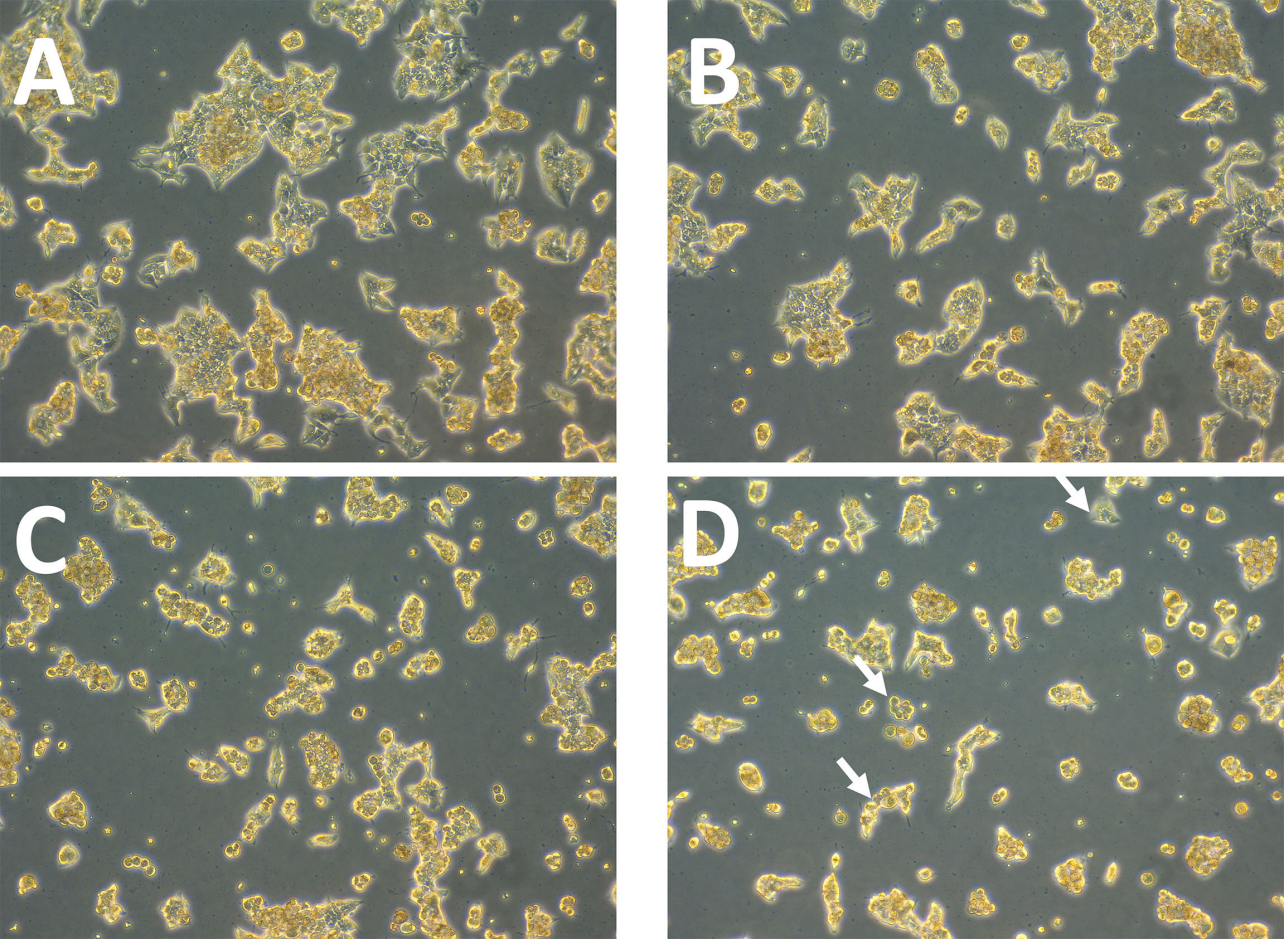
**(A–D)**. Effect of TZ and BMS-202 on ZR75 cell morphology. We note that treatment with **(B)** TZ and **(C)** BMS-202 alters cell morphology to a monolayer structure. **(D)** Combining both treatments increases cell-cell adhesion in a monolayer in comparison with the **(A)** control.

Afterwards, alterations in ZR75 cell morphology upon treatment with TZ and BMS-202, individually and combined were explored. ZR75 cells show round morphology, forming multilayer colonies as seen in untreated cells (**Figure 2A**). However, treatment with TZ and BMS-202 shifted cell morphology to a monolayer structure (**Figures 2B, C**). While, an increase in cell-cell adhesion in a monolayer after treatment with combination therapy was seen, with a lower number of cells (**Figure 2D**), consistent with our previous experiment.

**Figure 3:**
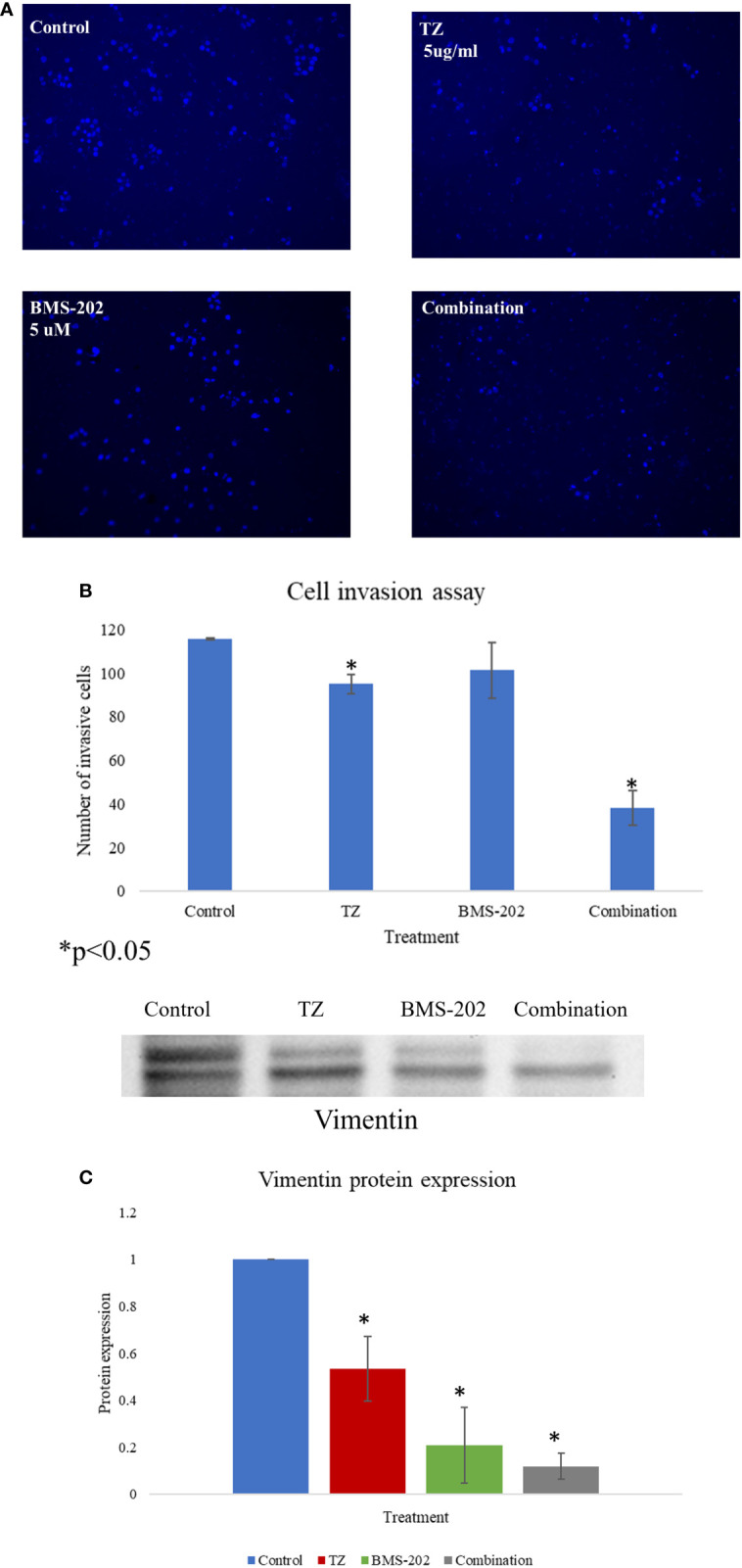
**(A–C)**. **(A)** The impact of TZ, BMS-202, and a combination of both on ZR75 cell invasiveness. **(A)** Compared to the control, both TZ and the combination therapy inhibit ZR75 cell invasion, with a more pronounced effect upon treatment with the combination therapy. **(B)** The number of invasive cells was quantified using ImageJ. **(C)** The changes in vimentin expression after treatment with TZ, BMS-202, and their combination. Data are presented as a percentage of the viable cells ± SEM.

Next, the impact of TZ, BMS-202 and their combination on cell invasion was investigated using Matrigel^®^ Invasion Chambers. Our data show a significant decrease in the number of invasive cells upon individual treatment with TZ but not with BMS-202. Interestingly, the combination therapy showed a more remarkable decrease in ZR75 cell invasiveness compared to monotherapy and the control (**Figures 3A, B**). To confirm our finding, we explored alterations in the protein expression of vimentin; a structural protein that plays important roles in cell-cell adhesion and cell invasiveness. We found a significant decrease in the protein expression, mostly in cells treated with the combination therapy of TZ and BMS-202 (**Figure 3C**).

**Figure 4:**
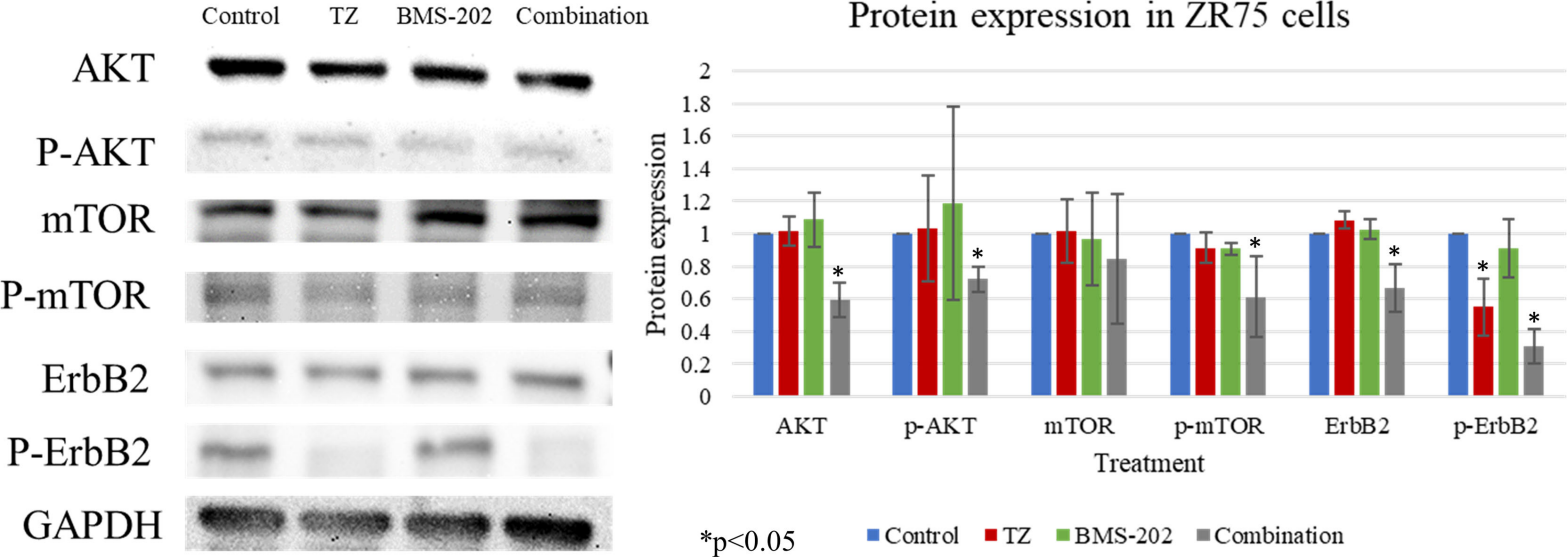
Western blot analysis of AKT, mTOR and ErbB2 in ZR75 cells under the effect of TZ and BMS-202. Treatment with both TZ and BMS-202 decreased the phosphorylation of ErbB2, AKT, and mTOR compared to individual treatment and untreated cells. GAPDH was used as a control for the amount of the loaded protein in this assay.

To gain further understanding of the molecular mechanisms of action of TZ and BMS-202 combination, we explored the expression patterns of key biomarkers critical in pathways related to growth, proliferation, differentiation, and other processes that contribute to cancer progression. Our data revealed that combining TZ with BMS-202 can significantly deregulate several pathways compared to individual treatment in ZR75 cells. For instance, the combination of TZ and BMS-202 decreased the phosphorylation of AKT and mTOR proteins significantly compared to individual treatment, where no such results were observed (**Figure 4**). In addition, the combination therapy decreased the phosphorylation of HER2, which is a major driver of HER2^+^ BC growth (**Figure 4**).

We then explored the effects of TZ and BMS-202 when used alone or in combination and quantified the growth inhibition of HER2^+^ BC cell colonies in soft agar.

**Figure 5:**
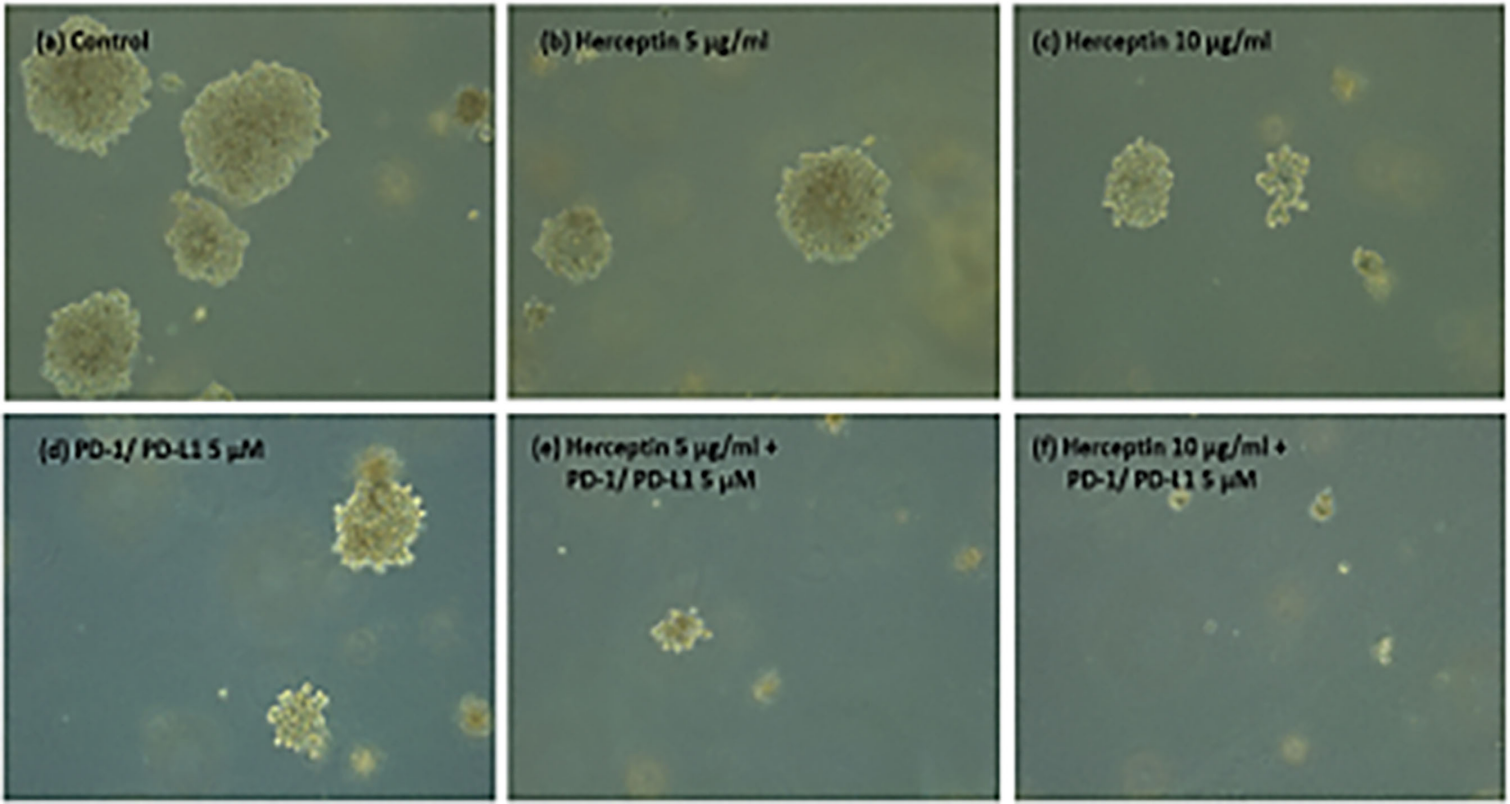
**(A–F)** ZR75 colonies imaged two weeks after treatment. Figure shows **(A)** Control **(B)** H5 **(C)** H10 **(D)** P5 **(E)** H5P5 and **(F)** H10P5 in order. There is a considerable reduction in the number of colonies and size of colonies when treated with combination of TZ and BMS-202.

**Figure 5** shows the images of the treated and untreated colonies after 14 days of plating.

**Figure 6:**
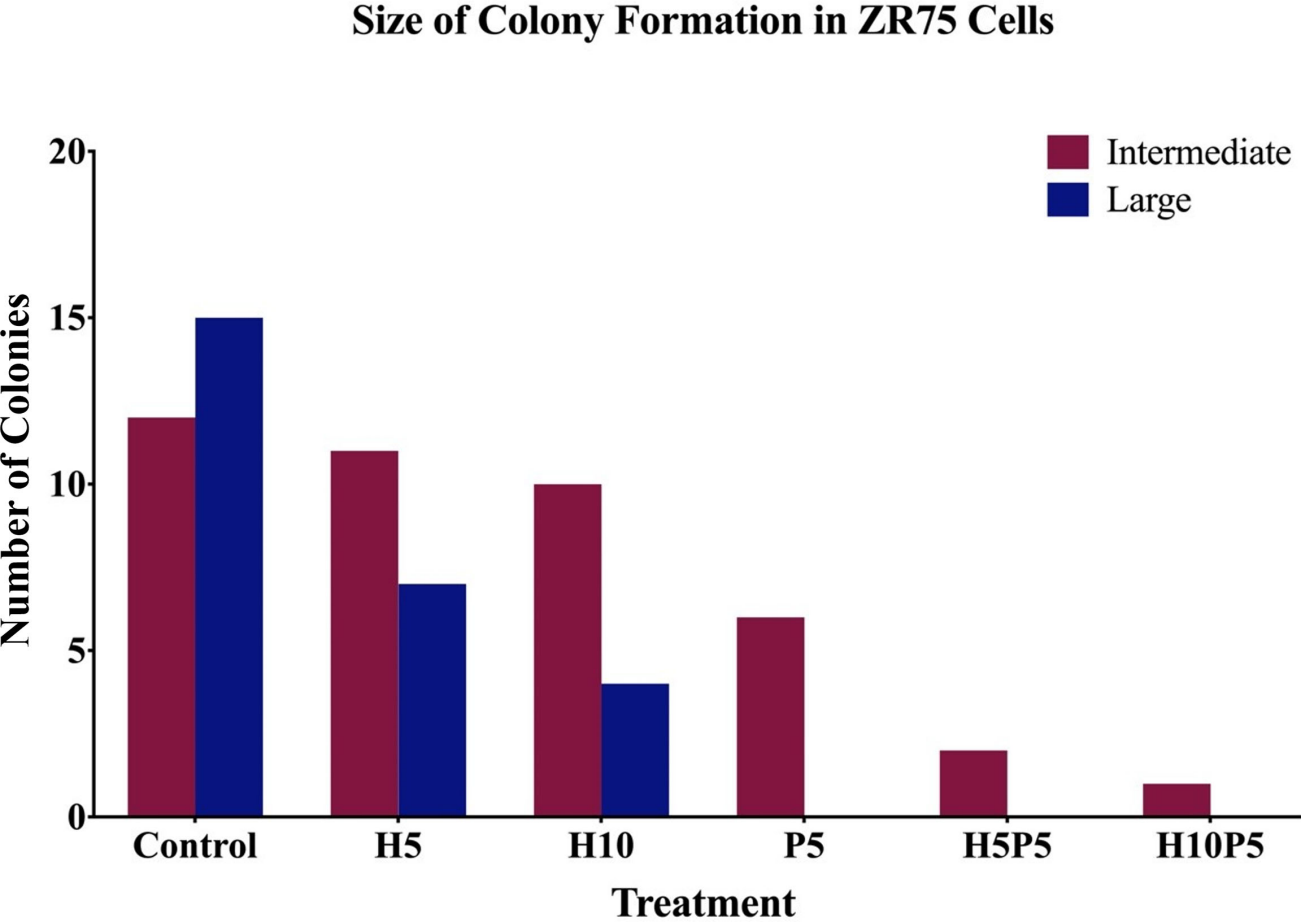
The number of big and intermediate colonies after 14 days of seeding in agar gel. It is shown that there is a considerable reduction in the number of colonies when treated with combination of TZ and BMS-202. Note that there are no big colonies in case of H5P5 and H10P5.

**Figure 6**. shows the average number of colonies in matched areas in each well for the control and treated cases. It can be seen that, while there is a considerable number of big colonies in the control case, all treated cases have either a lesser number or no big colonies. Notably, the wells treated with a combination of drugs (H5P5 and H10P5) have no big colonies at all. All these initial experiments with ZR75 cell lines point to the significant growth inhibition of HER2^+^ BC cells when combination drugs are used.

**Figure 7:**
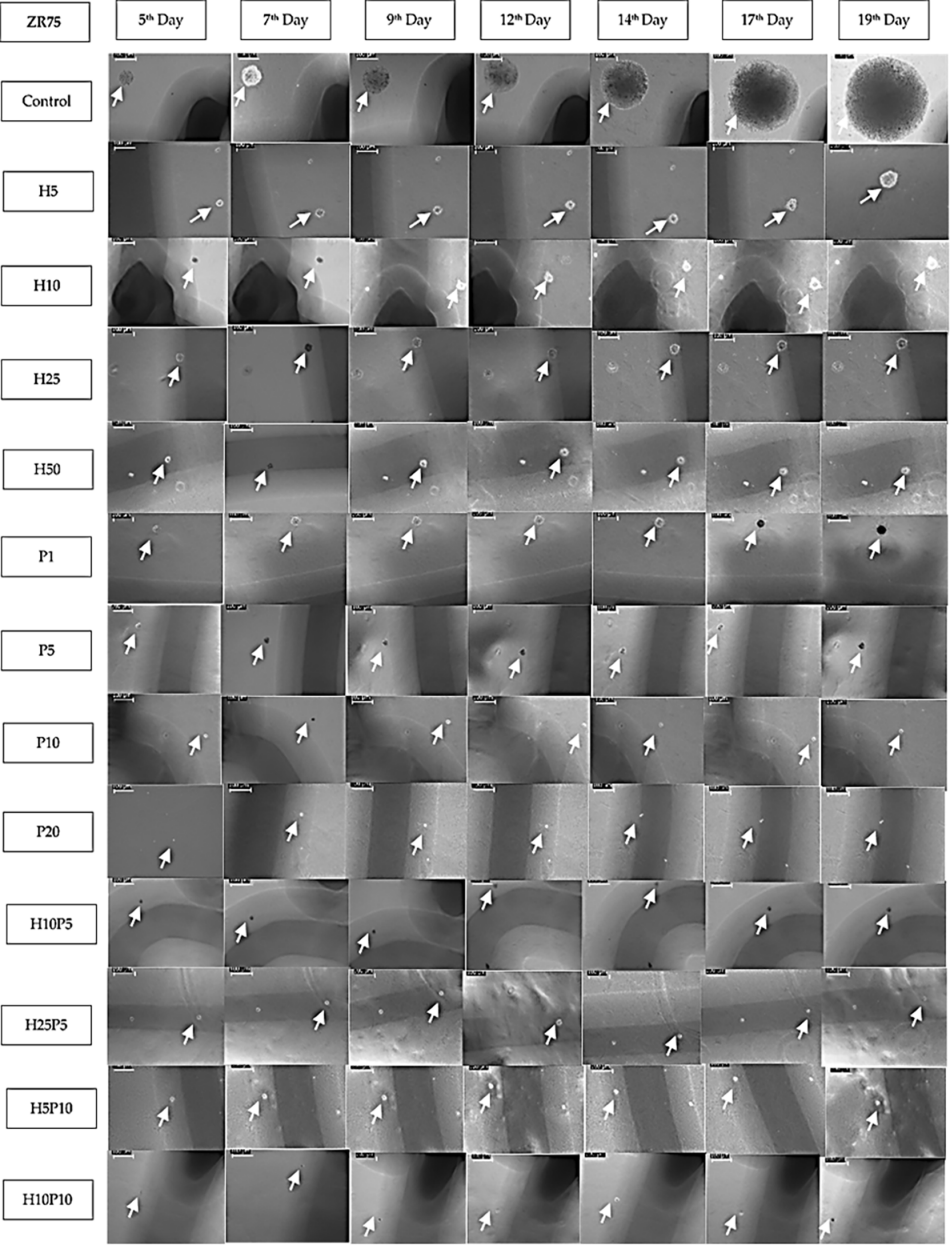
Images of ZR75 colonies (1 set) treated with various drug concentrations and combinations. Images are taken using an inverted microscope interfaced to LAS EZ software on 5th, 7th,9th, 12th, 14th, 17th, and 19th day after seeding. White arrow marks show the colonies. Images are calibrated (scale bar=100μm) using LAZ EZ software. Images for higher concentrations (H25P10, H25P20, and H50P20) are not shown as the growth inhibition is close to 100%. Shadows (dark line) of the markings made underneath the 6-well plate to track the colonies are also seen in most of the images.

As the preliminary experiments conducted revealed significant drug effect in the case of combined use of TZ and BMS-202 on HER2^+^ BC cells, we proceeded to collect time-series data to estimate the parameters for a mathematical model of cancer growth and drug-induced growth inhibition. In order to assess the efficacy of TZ and BMS-202 in the inhibition of colony formation of ZR75 cell lines, we quantified the growth of the same colonies over a period of time. To locate the same colony, markings were made under each well and the area of colonies were measured with images calibrated using LAS EZ software (**Figure 7**). Colonies with considerable change in size over the period of experiment (big colonies with more than 25 cells and intermediate colonies with 10 to 25 cells) were used for parameter estimation. However, in case of wells treated with drug concentration or combination that caused significant growth inhibition (e.g., P20, H25P10), there were only small, or no colonies left.

In general, exponential, logistic, Gompertz, Michaelis–Menten, Von Bertalanffy, and power-law models are used to represent tumor growth characteristics (2, 40, 41). Based on the comparison of various models for their descriptive power, identifiability, and predictability, the literature suggests that no single model is suitable for all types of cancers. Nevertheless, based on the extensive analysis reported in Benzekry et al., 2014, Sarapata et al., and summarized in Padmanabhan et al., 2020, the Gompertz model shows reasonable goodness of fit for cancers in breast, lung, head and neck, liver, bladder, and pancreas. In terms of best fit, power-law is ranked one, for most cancer types. Nonetheless, due to the biologically unjustifiable nature and high sensitivity of the power-law model to parameters, the Gompertz model or logistic model is preferred over the power-law model. In addition, the Gompertz model shows a good predictive ability for breast cancer data.

Out of many possible model options, we choose the Gompertz model as it has already proved to have reasonable fit and predictability with respect to BC data (37–39). The Gompertz model for BC cell colonies growth is given by


(1) $$\frac{\text{d}A\left(t\right)}{\text{d}t}=r\text{ln}\left(\frac{k}{A\left(t\right)}\right)A\left(t\right),A\left(0\right)=A_{0}$$

with the solution


(2) $$A\left(t\right)=ke^{\text{ln}\left(\frac{A_{0}}{k}\right)e^{-rt}}=k{\left(\frac{A_{0}}{k}\right)}^{{\text{e}}^{-rt}},$$

where *A(t)* is the area of the colony in µm^2^, *r* is the growth rate of the colony in days^-1^, and *k* is the carrying capacity of the environment in µm^2^. Gompertz model accounts for both the initial slow growth and saturation in growth towards the end due to space and nutrition (carrying capacity) constraints. **Table 1** shows values of *k, r*, and *A_0_* obtained by fitting the equivalent form of model (2) given by to the measured data, area of ZR75 colonies in agar assay, respectively. Model parameters were estimated using the trust-region-reflective algorithm in Matlab^®^. Specifically, an in-built function, namely, *lsqcurvefit()* which solves the nonlinear data-fitting problem in a least-squares sense were used to find the coefficients (*k, r*, and *A_0_*) that best fit the nonlinear function (2). See Appendix (Figs. A1-A18) for model fitting curves obtained using the Matlab^®^ algorithm.

**Table 1:** Gompertz model parameters for the growth of the ZR75 colonies in agar assay.

Set	No. of data set	*k* (mean (std. dev)) µm^2^	*A_0_* (mean (std. dev)) µm^2^	*r* (mean (std. dev)) days^-1^
Control	6	5.8e4 (4.8e4)	320.33 (183.85)	0.0911 (0.0880)
H5	7	8.49e8 (2.24e9)	202.247 (247.39)	0.1675 (0.0981)
H10	7	1.4e9 (2.43e9)	375.23 (162.41)	0.0443 (0.0515)
H25	7	1.3e9 (3.46e9)	127.04 (200.9)	0.288 (0.20)
H50	7	4.3e4 (5.2e4)	220.81 (170.80)	0.1562 (0.15)
P1	6	1.2e9 (2.2e9)	227.83 (211.70)	0.0651 (0.10)
P5	6	2.6e9 (3.1e9)	189.05 (152.64)	0.1586 (0.275)
P10	5	8.4e8 (1.0e7)	182.25 (63.98)	-0.259 (0.3)
P20	3	2.3e8 (4e8)	336.28 (241.84)	-0.038 (0.037)
H5P10	4	2.5e4 (4.9e4)	325.56 (45.62)	-0.2191 (0.29)
H10P5	6	3.3e8 (6.7e8)	224.88 (125.49)	-0.367 (0.4)
H10P10	6	1.4e8 (1.1e8)	–	–
H25P5	5	3.5e8 (5.8e8)	213.85 (122.73)	-0.06 (0.12)
H25P10	5	–	–	–
H25P20	4	–	–	–
H50P20	4	3.9e8 (7.8e8)	414.4 (101.21)	-0.03 (0.02)^1^

^1^P: PDL1 inhibitor (BMS-202), H: Herceptin (trastuzumab)

**Figure 7** shows one set of time-series data collected over 19 days which were used to quantify the growth of ZR75 colonies under treatment with various drug concentrations and combinations. As given in **Table 1**, up to 7 sets of such time-series data were obtained 2 or 3 days apart for parameter estimation. There was no colony formation at all in some of the wells (e.g., P20, H25P20).

From **Figure 7**, it can be seen that the growth rate is reduced for various treated cases compared to the control. However, the value of *r* in **Table 1** does not reflect this growth inhibition, this is due to the fact that the nonlinear least-squares algorithm allows the variables *k, r*, and *A _0_* to vary appropriately to find an exact fit to the time-series data. Hence, in order to quantify the growth inhibition due to treatment, the Gompertz model is rewritten as


(3) $$\frac{\text{d}A\left(t\right)}{\text{d}t}=\left(r-a\right)\text{ln}\left(\frac{k}{A\left(t\right)}\right)A\left(t\right)$$

with the solution


(4) $$A\left(t\right)=k{\left(\frac{A_{0}}{k}\right)}^{{\text{e}}^{-\left(r-a\right)t}}$$

where *a* models the drug effect, that is the per day growth inhibition due to treatment.

Here, note that the input data is the area of the colonies, using which we derived the growth rate, carrying capacity, and drug effect. As the experiment is conducted in agar gel, on day zero (cell seeding day) the cells were not at all visible in the images, hence curve fitting is conducted using measured area available from 5^th^ day of seeding. The parameter values shown in **Table 1** do not directly reveal the difference in growth inhibition caused by different drug concentrations or combination because of the variability in *a, k*, and ***A_0_***. However, from **Figure 7** it is clear that, there is significant growth inhibition in treated colonies compared to the control. For instance, comparing control and H5, when the area of colonies in the control wells was in the range 1000-7500 µm^2^ that of H5 was only in the range 250-2250 µm^2^(**Figures A1, A2** in **Supplementary File**. Hence, there is a significant reduction in the growth rate in the case of H5. However, due to difference in initial condition (on Day 5) and the wide range of areas of different colonies each day, plotting a single interpolated curve from all replicates did not lead to a conclusive result. Hence, to show the growth pattern in each treatment case and thereby quantify the growth inhibition, we decided to plot the growth curve of each colony separately. As shown in Figures A1-A10 in the **Supplementary File**, Matlab’s *lsqcurvefit()* has successfully derived best-fit parameters, however, as mentioned earlier this significant growth inhibition is not reflected in the value of *r* given in **Table 1**. This is because, we estimated 3 parameters required for fitting the nonlinear curve such as *r*, *k* and ***A_0_***. Hence, to have a clear comparison between the growth inhibition of various drug concentrations and combinations, we fixed two values (*k* and ***A_0_***), and re-estimated the growth of control set alone (*r_c_*), then, using *r_c_* in equation (4), we estimated the *a* (growth inhibition*)* value for each drug concentration and combination. This is a valid assumption as we used uniform cell seeding density and supplied the same amount of cell culture media to all wells throughout the experiments.

Next, the rationale behind the choice of the value of ***A_0,_*** is mentioned in **Table 2**. As shown in **Figure 7**, we started measuring the area of colonies on the 5^th^ day of seeding i.e. when the colonies were visible. Using the measured data, the fitting algorithm was used to predict the initial area (***A_0_***), the carrying capacity (*k*), and the growth rate (*r*). In order to perform a comparative assessment of the change in growth inhibition between the control and various treated cases, rather than determining the values of ***A_0_*** and *k*, we fixed these two parameters for all the cases and re-estimated the value of growth inhibition, *a*, alone. For instance, the initial area ***A_0_*** of the colony estimated by the algorithm varied within the range 127.04-414.40 µm^2^ for 88 sets in **Table 1**). Hence, we fixed the value of ***A_0_*** as 200 um^2^. We chose a value closer to the lower range limit since fixing ***A_0_*** greater than the measured value on day 5 would result in negative growth rates for cases with significant growth inhibition (e.g. P20). The value of the carrying capacity (*k*) estimated by the algorithm varied from 2.5e^4^ – 2.6e^9^ µm^2^ for 88 sets in **Table 1**).

**Table 2:** Drug induced growth inhibition of ZR75 colonies in agar assay.

Set	No. of data set	Drug effect (*a*) days^-1^, (mean (std. dev.))	Growth inhibition (%)
Control	6	0	0
H5	7	0.0081 (0.0026)	33.75 (5.4)
H10	7	0.0109 (0.0054)	45.42 (11.2)
H25	7	0.0055 (0.0032)	22.92 (6.6)
H50	7	0.0053 (0.0071)	22.09 (14.7)
P1	6	0.0120 (0.0058)	50 (12.0)
P5	6	0.0129 (0.0062)	53.75 (12.9)
P10	5	0.0236 (0.0019)	98.34 (3.9)
P20	3	0.0535 (0.0214)	100*
H5P10	4	0.0200 (0.0023)	83.34 (4.7)
H10P5	6	0.0224 (0.0030)	93.34 (6.2)
H10P10	6	0.0224 (0.0055)	93.34 (11.4)
H25P5	5	0.0225 (0.0046)	93.75 (9.5)
H25P10	5	0.0225 (0.0056)	93.75 (11.6)
H25P20	4	0.0315 (0.0073)	100*
H50P20	4	0.0482 (0.0183)	100*

The drug effect parameter a is estimated using model (4) by fixing k=1e6 µm2, A0 = 200 µm2, and the growth rate of the control is set as r=0.0240 (0.0042). The overall growth rate of treated colonies is rtreat =r-a and growth inhibition is calculated as % GI=(1-(rtreat/r)) ×100. * Note that while calculating GI value for P20, H25P20, H50P20, as value of r<a, r-a becomes negative resulting in %GI>100, which is rounded off to 100%.

Next, the rationale behind the choice of *k*. Considering space limitation of a single well (34.8 mm diameter, area 3802.66 e^6^ µm^2^ and seeding density of 1000 cells/well, each colony can have a maximum area of 3.8 e^6^ µm^2^. Hence, we fixed carrying capacity ***A_0_*** as 1 e^6^. We tested the algorithm by fixing different reasonable values of ***A_0_*** and *k* and in all cases, as expected (due to uniform cell seeding and well size), there is negligible variance in the estimated value of *a* (cases 1 and 2 in **Table AT1 in the Supplementary File**). Moreover, as shown in **Figure 5**, small, intermediate, and big colonies were seen in agar assay, hence heterogeneity in the colony size is expected. We excluded very small colonies and used images with intermediate and big colonies. However, even after including both big and intermediate colonies, as shown in **Tables 2** and **3** a trend of increased drug effect is seen in the case of combination data.

**Table 2** shows the results obtained for ZR75. The overall growth rate of treated colonies is given by *r*_treat_ =*r-a*, using *r*_treat_ the percentage value of growth inhibition (GI) in each case is calculated as % GI=(1-(*r*_treat_/*r*))×100, where *r*_c_ is the mean growth rate of the control data set estimated by fixing the values of *k* and A_0_. To summarize, the steps involved in generating **Table 2** are: (1) Fix values for *k* and A_0_ and estimate the growth rate (*r*_c_) of control data set, (2) Set *r=r*_c_ in equation, (3) and estimate the value of growth inhibition parameter (a) for each data set. From **Table 2**, it can be seen that BMS-202 can cause dose-dependent growth inhibition of ZR75 colonies. The % GI of ZR75 colonies are 50%, 53.75%, 98.34%, and 100% for P1, P5, P10, and P20, respectively. Moreover, a combination of TZ and BMS-202 resulted in increased growth inhibition of ZR75 colonies compared to respective monotherapies. For instance, %GI for H10P5 was 93.34%, whereas for H10 and P5%GI was 45.42% and 53.75%, respectively. It can also be seen from **Table 2** that all combination therapy concentrations resulted in at least 80% GI of ZR75 colonies. Note that these results are for an immune deprived environment. Hence, a synergistic drug combination effect is expected in an immune-competent *in vivo* environment which will have additional effector cell-mediated cytotoxicity as well.

## Discussion

It is well known that the mechanism of action behind many of the anti-HER2 agents (trastuzumab, pertuzumab, trastuzumab emtansine, margetuximab, etc.) involve immune effector modulation (10, 31, 42). Moreover, the significant correlation between the presence of TIL (tumor-infiltrating leukocytes) in the tumor microenvironment (TME) and improved survival rate says why disintegration of the immune evasion strategy of cancer cells using ICB is an idea worth exploring for HER2^+^ BC in particular (2, 27, 43, 44). An interesting study revealed that PD-L1 expression was significantly increased when treated with TZ in HER2-amplified gastric cancer cell lines co-cultured with peripheral blood mononuclear cells (PBMCs). Another study shows that TZ sensitive HER^+^ BC reportedly express higher levels of PD-L1 than TZ insensitive BC cells (26). Hence, additional use of ICBs can restore T-cell augmentation and thus enhance antibody-mediated cytotoxicity of TZ. Pre-clinical results report synergy in action when TZ is used with ICB-based (anti-PD-1/anti-CD137 mAb) therapy (45). A combination therapy using margetuximab (anti-HER2) and pembrolizumab (anti-PD-1) showed acceptable safety and tolerability with no dose-limiting toxicities in HER2^+^ gastro-esophageal adenocarcinoma (32). Similarly, our study reveals that the combination therapy using TZ (anti-HER2, mAb) and BMS-202 (anti-PD-1/PD-L1, SmI) results in improved growth inhibition compared to monotherapies even in an immune cell deprived environment, as shown in contingency **Table 3** for % growth inhibition of ZR75 colonies when treated with various drug concentrations and combinations. All these studies serve as a proof of concept for expected synergistic anti-tumor activity in the combination of anti-HER2 and anti-PD-1 agents in an immunocompetent *in vivo* environment (32, 46).

**Table 3:** Contingency table showing % growth inhibition of ZR75 colonies when treated with various drug concentrations and combinations.

% Growth inhibition P alone	Conc.	% Growth inhibition with combination treatment			
100*	P20	-	-	100	100
98.34*	P10	83.34	93.34	93.75	-
53.75*	P5	-	93.34	93.75	-
50*	P1	-	-	-	-
	Conc.	H5	H10	H25	H50
	% Growth inhibition H alone	33.75*	45.42*	22.92*	22.09*

Values given in bold indicate % Growth inhibitions for combination therapy and those with * are for monotherapy.

Many mAbs including pembrolizumab and durvalumab, which were FDA approved for many other cancers, are currently under investigation for HER2^+^ BC particularly to evaluate dose-limiting toxicities, maximum tolerated dose (MTD), recommended phase-II dose (RP2D), and objective response (OR). In a phase 2 trial (PANACEA, pembrolizumab + TZ), it is reported that when 15% (6/40) of PD-L1^+^ cases achieved OR, none of the PD-L1^-^ achieved OR. During the 13·6 (for PD-L1^+^ tumors) and 12·2 (for PD-L1^-^ tumors) months evaluation period, even though grade 3-5 adverse events (AE) were reported in 50% of patients (with treatment discontinuation due to AE in 8% of the patients), the overall findings suggest that the combination of pembrolizumab and TZ is safe to use and showed continuing clinical benefits in HER2^+^ BC patients with TZ-resistant and PD-L1^+^ tumors (47). On a scale of 5, adverse effects in grades 1-2 were reported, RP2D is a full dose of durvalumab and TZ, and no safety issues were reported (25). Other currently ongoing clinical trials include NCT03417544 (atezolizumab, pertuzumab, TZ, HER2^+^ MBC), NCT03125928 (atezolizumab, paclitaxel, TZ, pertuzumab, HER2^+^ MBC), NCT03595592, (TZ, pertuzumab, carboplatin, paclitaxel, atezolizumab, HER2^+^, locally advanced BC), and NCT03199885 (paclitaxel, TZ, pertuzumab, atezolizumab, for HER2^+^ MBC). Even ICB-based DNA vaccines are under clinical trials for managing HER2^+^ cancers (48). However, note that in PANACEA only 15% OR is reported which means that we are quite far from figuring out a therapy that ensures 100% complete response or relapse-free survival for HER2^+^ BC patients (28, 49).

As mentioned earlier resistance to mAb-based therapy and relapse after treatment that were reported in earlier cases calls for more research using other drug modalities such as SmIs, peptides, and macrocycle. BMS-202 is a biphenyl SmI developed by Bristol Myers Squibb (BMS) which can stabilize PD-L1 protein dimers (36, 50). Specifically, BMS-202 can dive deep into the hydrophobic cylindric pocket created by two juxtaposed PD-L1 molecules and stabilize and hide away a PD-L1 homodimer, and thus prevent it from interacting with a PD-1, blocking intracellular signalization which leads to immune evasion of cancer cells (33). Biophysical and crystallographic studies suggest that BMS-202 can inhibit the interaction of the PD-1 receptor with its ligand by facilitating the dimerization of the latter (29, 37, 51–53). Anti-tumor activities and immunomodulatory effects of BMS-202 is studied using *in vitro* (human CD3^+^ cells) and *in vivo* studies; BMS-202, PD-1/PD-L1 binding is blocked leading to increased IFN-γ secretion *in vitro* (36). Similarly, *in vivo* experiments showed increased IFN-γ levels, cytotoxic T cells, and reduced T regulatory cells in blood (36). Due to the advantages of SmIs over mAbs, there is an increased interest in understanding the usefulness of BMS-202 in treating various cancers (27, 33–35). Study by Zhang et al. (53),, BMS-202 entrapped in nanoparticles (BMS-202 NPs) were used in a BC mice model (4T1 tumor-bearing mice) to study tumor deliverability and anti-cancer activity of BMS-202 NPs. This study showed the impressive anti-tumor and anti-metastatic effects of BMS-202 NPs (53).

*In-vitro* experiments reveal that BMS-202 can inhibit the proliferation of PD-L1^+^ SCC-3 cells (IC50 15 μM) and anti-CD3 antibody-activated Jurkat cells (IC50 10 μM) (52). As per this study, BMS-202 does not regulate the expression of PD-1/PD-L1 on cells, rather it inhibits the formation of the PD-1/PD-L1 complex by facilitating the dimerization of PD-L1 (52). Most importantly, BMS-202 showed a clear and direct anti-tumor effect against SCC compared to control in severely immune-deficient (MHC-double knockout) NOG mouse (52). The study using PD-L1^+^ SCC-3 cells *in vivo* (in NOG mouse) indicate that the antitumor activity of BMS-202 might be partly mediated by immune modulation and partly by the off-target cytotoxic effect (52). In line with these findings, our results also indicate that the anti-tumor activity of BMS-202 on HER2^+^ BC cells is partly by the off-target cytotoxic effect. More *in vitro* and *in vivo* studies are required to substantiate the synergy in action when BMS-202 is used along with TZ. Note that both drugs increase the level of cytokine interferon in the tumor microenvironment (TME). Another question that remains is whether T cell exhaustion in the TME will limit or saturate the overall efficacy when two drugs are used together *in-vivo*.

The role of vimentin in cancer cell motility, migration and invasion is well established (54). It is a major mediator in the epithelial-mesenchymal transition event, which results in cancer dissemination and metastasis (54, 55). Furthermore, knocking out vimentin attenuates tumor cell invasion (56). This highlights the importance of vimentin as a potential target to inhibit tumor progression. In this study, we revealed that vimentin protein levels were significantly decreased upon treatment with the combination of TZ and BMS-202. Accompanied with cell invasion data as well as the deregulation of AKT, mTOR and HER2, which play an important role in carcinogenesis (refs), we suggest that the combination therapy of TZ and BMS-202 may serve as an inhibitor of HER2+ breast cancer cell invasion.

HER2 amplification in HER2+ cancers is considered the major driver of tumor growth and progression. Upon dimerization, HER2 autophosphorylation activates several downstream molecular pathways, such as PKC and AKT/mTOR (57). These pathways control essential biological processes that can work in the favor of cancer cells when deregulated. These processes include cell survival and proliferation, motility, invasion, and differentiation. This shows why targeting HER2 with anti-HER2 drugs or monoclonal antibodies is essential in the management of HER2+ cancers (58). We herein report that treatment with TZ and BMS-202 for 48 hours can suppresses the expression of HER2 receptor, while mostly affecting its phosphorylation. In addition, we noticed a deregulation in the expression patterns of AKT/mTOR upon treatment, which was more pronounced when we used the combination of TZ and BMS-202.

In general, there is a strong indication of the synergistic outcome when anti-HER2 and ICB-based therapies are applied together (17, 21, 22, 27, 28, 59). When it comes to combination therapy, along with empirical experiments, mathematical models can be used to evaluate effective dose combinations and order of treatment (2, 60, 61). Study by Jarrett et al. (61), demonstrated an experimentally-driven mathematical model is used to analyze combination therapy (TZ+paclitaxel) protocols for HER2^+^ BC. Another mathematical model-based analysis reveals TNF-α induced reduction in drug-resistance to anti-PD-1 (62). Similarly, a mathematical model was developed to represent combination therapy (cancer vaccine and ICB) (51). Thus, it is obvious that mathematical models, if properly devised with appropriate measurable biomarkers can be used to conduct risk-free, cost-effective *in silico* analysis to identify patient cohorts that will benefit from a certain type of treatment (63, 64).

The contribution of this paper comes in many folds. We herein present (1) a feasible methodology to use agar-assay based colony formation experiments to track the growth of the same colony over a period of time and to build a mathematical model based on the time-series data derived (2). Our data revealed improved growth inhibition of colonies in the case of combination treatment compared to single agent cases (3), The Gompertz model is validated as a suitable model to describe the growth pattern of breast cancer cell lines, and (4) the combination treatment with TZ and BMS-202 decreased the cell’s invasiveness along with altering several key pathways, such as AKT/mTOR and ErbB2 compared to monotherapy. The application of the mathematical model discussed in this paper is limited to the study of growth patterns of breast cancer cell lines, drug-induced percentage growth inhibition, and combination drug effect. Herein it is important to highlight that a single term Gompertz model is inadequate to reflect the complex dynamics in the tumor microenvironment *in vivo*, which involves the interaction of multiple cells and biochemicals (such as crosstalk between normal, cancer, endothelial and immune cells as well as cytokines, chemokines etc.). Complex models with multiple terms where each term can be linear (such as Gompertz, power law, logistic model) may predict cancer behavior in future timescale as each term in the model equation accommodate (1) growth (2) competition between cells (3) cell differentiation/mutation and (4) the effect of therapy, for each cell type in the tumor microenvironment. However, in this paper, we have used Gompertz model to represent treatment induced growth inhibition alone, not the complete dynamics of a tumor microenvironment. More complex experiments that involve cell-coculture (breast cancer cells with peripheral blood mononuclear cells (PBMCs)) can be used to mimic a tumor microenvironment and thus build more complex mathematical models that can be used to derive critical information regarding immune cell-induced enhancement and saturation of drug effect due to T cell exhaustion. More importantly, we envisage that the results discussed in this paper will lead to more studies that investigate molecular pathways, if any, that improve the potency of TZ when used along with BMS-202 in HER2 treatment.

In this paper, we present a Gompertz model-based method to quantify drug-induced growth inhibition. Development of similar mathematical models which represent the dynamics of HER2^+^ BC cells, immune cells, and drugs involved are interesting directions for future research. Such models can be used to evaluate the critical threshold of T cell exhaustion that will hinder a patient from getting the potential benefits expected out of ICB-based therapy (16, 65). Apart from the ZR-75 results reported in this paper, we have conducted a similar study using the SKBR3 cell line (please refer to **Supplementary Data**) wherein the Gompertz model exhibited good fit, however, with slightly different values for variables (r, k, a). Hence, investigating how far we can generalize the model parameters for various cell lines can be an interesting direction for future work. Similarly, deriving a mathematical function that fits the measured growth inhibitions with respect to the two different drug doses used (**Table 3**) is also desirable for identifying the best dosing combination. In short mathematical model-based approaches can act as a link to facilitate the integration of multiple computational strategies towards tailoring personalized treatment protocols by accommodating patient-specific characteristics (1, 3, 30, 63, 66, 67). Specifically, investigations based on computational approaches which can quantify indications of diagnostic, therapeutic, and prognostic biomarkers pertaining to HER2^+^ BC can accelerate drug development, drug repositioning, and identification of effective drug combination for managing the disease (2, 68–70).

## Conclusions

In order to have a realistic assessment of cancer disease prognosis and predictive outcomes, biomedical research frameworks must adopt more quantitative methods to gain insight on disease mechanisms, therapy options, and prognostic features of biomarkers. The significant correlation between immune response, PD-1/PD-L1 expression, and disease prognosis of HER2^+^ BC indicates that tailored ICB-based therapies can improve the management of HER2^+^ BC patients. Our mathematical model-based study points out that the combination therapy using trastuzumab (anti-HER2, mAb) and BMS-202 (anti-PD-1/PD-L1, SmI) results in a significant growth inhibition of HER2^+^ BC cell lines compared with monotherapies even in an immune cell deprived environment. Nevertheless, further investigations are imperative to uncover the potential crosstalk between PD-1/PD-L1 inhibitors and HER2 growth signaling pathways in breast cancer.

## Data availability statement

The original contributions presented in the study are included in the article/**Supplementary Material**. Further inquiries can be directed to the corresponding authors.

## Author contributions

Conceptualization, N.M. and A-EM. Data curation, RP and HK. Formal analysis, RP and HK. Funding acquisition, SV. Methodology, RP and HK. Project administration, A-EM. Resources, A-EM. Supervision, NM and A-EM. Writing – original draft, RP, HK, IG and A-EM. Writing – review and editing, RP, HK, IG, NM, AH, SV and A-EM. All authors contributed to the article and approved the submitted version.

## Glossary

**Table:**

ATCC	American type culture collection
BC	breast cancer
BMS	Bristol Myers Squibb
CTLA	cytotoxic T-lymphocyte-associated protein
DFS	disease-free survival
FBS	fetal bovine serum
GI	growth inhibition
HER	human-epidermal growth factor receptor
ICB	immune checkpoint blockade
IFN	interferon
irAEs	immune-related adverse events
mAbs	monoclonal antibodies
MHC	major histocompatibility complex
MTD	maximum tolerated dose
NOG	severely immunodeficient mouse
NP	nanoparticle
OR	objective response
PBS	phosphate buffered saline
PD-1	programmed death receptor
PD-L1	programmed death receptor ligand
PI	propidium iodide
RP2D	recommended phase-II dose
RPMI	Roswell Park Memorial Institute
SCC	squamous cell carcinoma
SmIs	small molecule inhibitors
TIL	tumor-infiltrating leukocytes
TME	tumor microenvironment
TNBC	triple-negative breast cancer
TNF	tumor necrosis factor
ICB	Immune checkpoint blockade
HER	human epidermal growth factor receptor
BC	breast cancer
TZ	trastuzumab
