# Supplementary material for: Quantification of the growth suppression of HER2+ breast cancer colonies under the effect of trastuzumab and PD-1/PD-L1 inhibitor
Source: Front Oncol. 2022 Dec 21;12:977664. doi: 10.3389/fonc.2022.977664 (PMC9769711; doi:10.3389/fonc.2022.977664)
Supplement: Supplementary file 1 [file DataSheet_1.pdf]

Supplementary file

Agar assay-model fitting ZR75

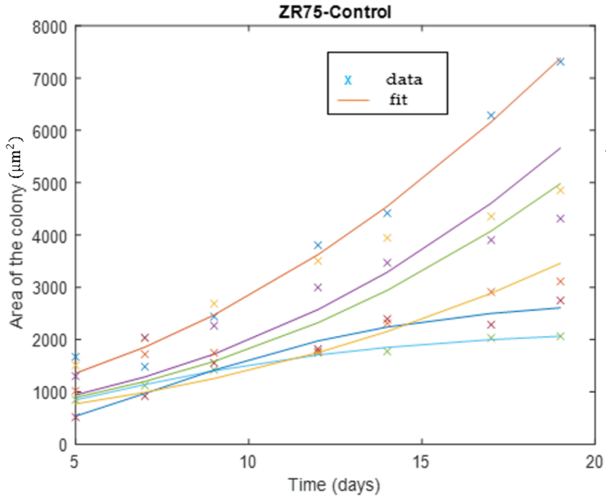

Figure A1: Model fitting curves for ZR75, 6 sets of control data

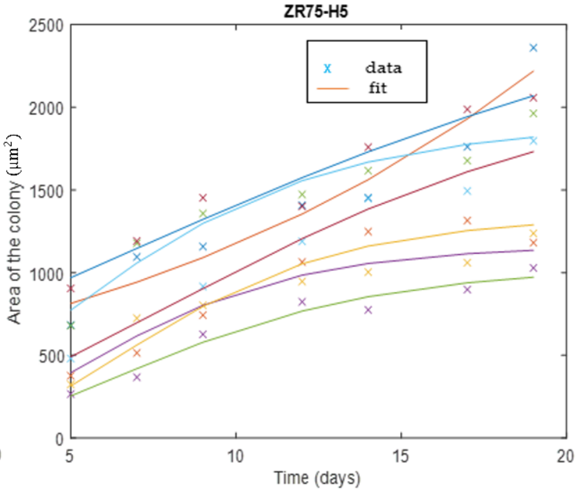

Figure A2: Model fitting curves for ZR75, 7 sets of H5 data

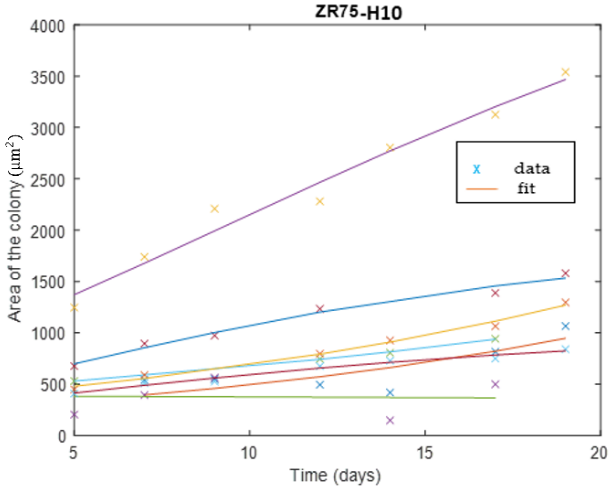

Figure A3: Model fitting curves for ZR75, 7 sets of H10 data

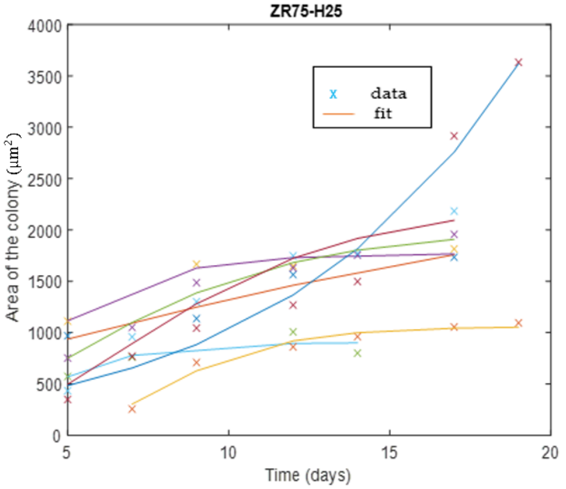

Figure A4: Model fitting curves for ZR75, 7 sets of H25 data

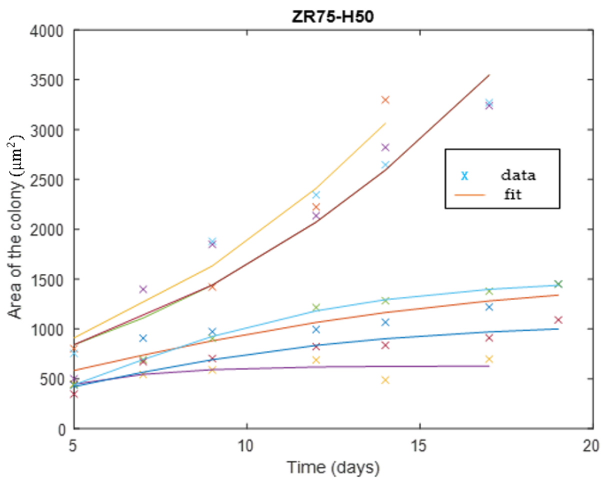

Figure A5: Model fitting curves for ZR75, 6 sets of H50 data

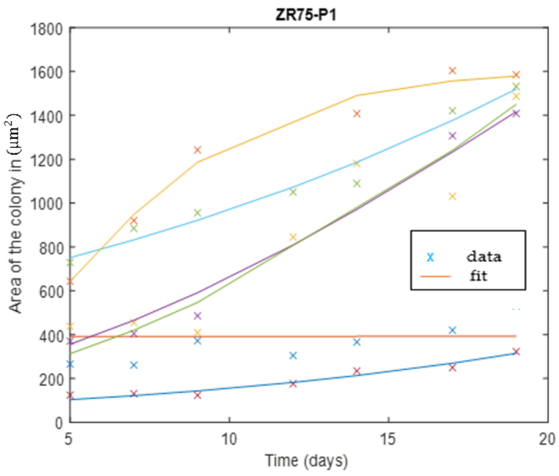

Figure A6: Model fitting curves for ZR75, 6 sets of P1 data

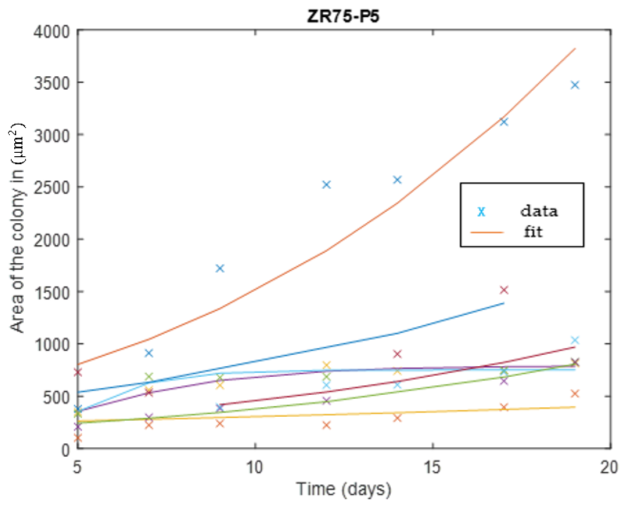

Figure A7: Model fitting curves for ZR75, 7 sets of P5 data

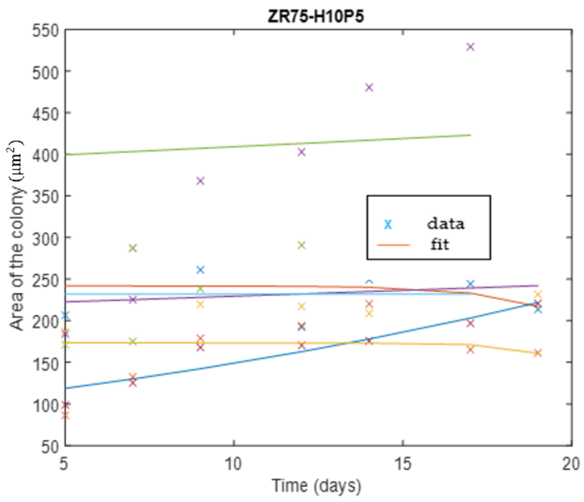

Figure A8: Model fitting curves for ZR75, 6 sets of H10P5 data

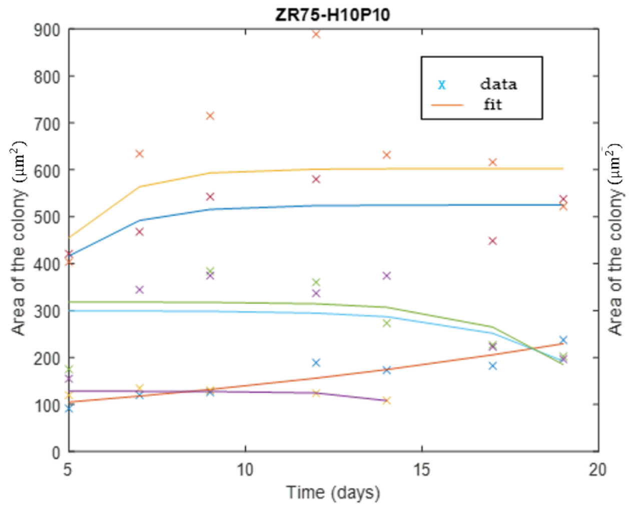

Figure A9: Model fitting curves for ZR75, 6 sets of H10P10

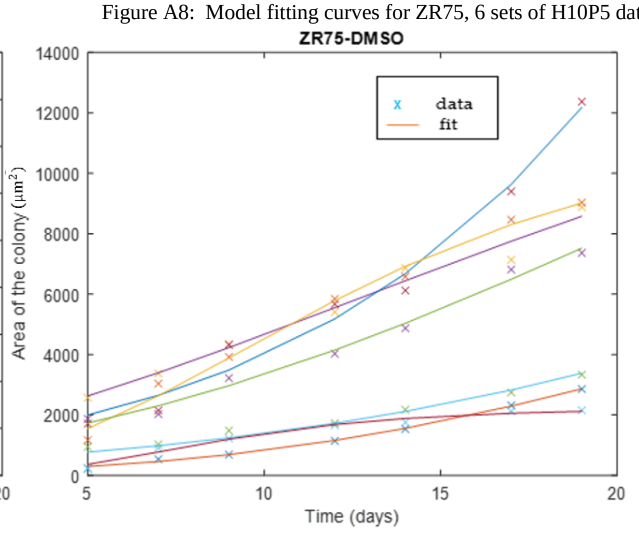

Figure A10: Model fitting curves for ZR75, 7 sets of DMSO data

Table AT1: Drug induced growth inhibition of ZR75 colonies in agar assay. The drug effect parameter *a* is estimated using model (4) for two cases, case (1) by fixing k=1e6 μm^2, A_0=200 μm^2, and the growth rate of the untreated (control) colony is set as r_c=0.0240, case (2) by fixing k=1e6 μm^2, A_0=10 μm^2, and the growth rate of the untreated (control) colony is set as r_c=0.0404. The overall growth rate of treated colonies is r_{treat} =r-a and growth inhibition is calculated as % GI=(1-(r_{treat}/r_c)) ×100.

Set	No. of data set	Drug effect (*a*) days^{-1}, (mean (std. dev.))	Drug effect (*a*) days^{-1}, each curve, case 2
Control	6	-0.00612749342352310	-0.00633218849191378
		-0.00267914705080568	-0.00262532738193124
		0.00518832147421401	0.00516324219703852
		0.00330672467322264	0.00316569693054745
		0.00201060217408234	0.00188575209312794
		-0.00141541001183606	-0.00140278752371447
		**Mean= 4.72663058923579e-05**	**Mean= -2.42686961409293e-05**
		**Std dev= 0.00420974142534772**	**Std dev= 0.00422350569968956**
H5	7	0.00557241329006354	0.00523587790143663
		0.0103787600348873	0.0100269974906805
		0.00617613652661463	0.00608246306281112
		0.00543814210536747	0.00535381989704300
		0.00966894095361044	0.00941646058425729
		0.0121460422005404	0.0115707911823364
		0.00747210880646278	0.00712728125834817
		**Mean= 0.00812179198822094**	**Mean= 0.00783052733955902**
		**Std dev= 0.00263284182042992**	**Std dev= 0.00250818022425086**
H10	7	0.0132809131513060	0.0123257008860133
		0.000688836133061182	0.000669988971116384
		0.0114954490239165	0.00914037602731365
		0.00834363014481328	0.00843827825821444
		0.0106507450245906	0.0101328933864437
		0.0182041804214405	0.0157558941726825
		0.0134269497393157	0.0129210989499048
		**Mean= 0.0108701005197777**	**Mean= 0.00991203295024125**
		**Std dev= 0.00542553527516648**	**Std dev= 0.00478300025480849**
H25	7	0.00521528483456787	0.00384068688694981

		0.00436239893961209<br>0.00874248281564587<br>0.00228408652717466<br>0.0111884008688486<br>0.00372504903791874<br>0.00320657190775880<br>**Mean= 0.00553203927593238**<br>**Std dev= 0.00324016397071860**	0.00322862204249973<br>0.00317711229720570<br>0.00163037140671406<br>0.0107130324190371<br>0.00177888258213873<br>0.00107750943381074<br>**Mean= 0.00363517386690799**<br>**Std dev= 0.00327923059226710**
H50	7	0.00920018666597362<br>0.0136966902433290<br>0.00856826827290443<br>0.0115943336437705<br>-0.00436486717765257<br>-0.000904384428196315<br>-0.000838334632684921<br>**Mean= 0.00527884179820626**<br>**Std dev= 0.00713671421436876**	0.00888994089151760<br>0.0113142769875071<br>0.00833579584397318<br>0.0111631040545341<br>-0.0106381725154331<br>-0.00295370381380634<br>-0.00289219177118301<br>**Mean= 0.00331700709672993**<br>**Std dev= 0.00870235982038088**
P1	6	0.0181681968064486<br>0.00991391650038746<br>0.00852680974257265<br>0.0230361280344447<br>0.00774395628354122<br>0.00999974027704771<br>**Mean= 0.0128981246074071**<br>**Std dev= 0.00622145881575175**	0.0169856339061427<br>0.00926764730718005<br>0.00823120170043477<br>0.0206674599451957<br>0.00774682379924620<br>0.00982154259513312<br>**Mean= 0.0121200515422221**<br>**Std dev= 0.00537400613008875**
P5	6	0.0132720402715241<br>0.0134135308000528<br>0.00839651129582386<br>0.0193631717761220<br>0.0149164172739646<br>0.0133395482080111<br>**Mean= 0.0137835366042497**<br>**Std dev= 0.00352225630601051**	0.0129178542816114<br>0.0134105893206017<br>0.00624193918365589<br>0.0175805148272640<br>0.0142018990794279<br>0.0124675656224005<br>**Mean= 0.0128033937191602**<br>**Std dev= 0.00369659186751847**
P10	5	0.0244672999311330	0.0232824418432795

		0.0248417274572760	0.0230446446476233
		0.0221958970246248	0.0209217608463690
		0.0254854030949932	0.0228518544510777
		0.0209476908419471	0.0208032266583806
		**Mean= 0.0235876036699948**	**Mean= 0.0221807856893460**
		**Std dev= 0.00192706524083043**	**Std dev= 0.00121377966527034**
P20	3	0.0407224288642159	0.0307527371366993
		0.0785370278248220	0.0405507362684397
		0.0423221463471105	0.0318665164589092
		**Mean= 0.0538605343453828**	**0.0343899966213494**
		**Std dev= 0.0213854336368677**	**0.00536434160396861**
H5P10	4	0.0209069780561113	0.0216143399111566
		0.0167942389301544	0.0202060346672416
		0.0105278244402237	0.0166782631303704
		0.0185489904973403	0.0214252422486852
		**Mean= 0.0166945079809574**	**Mean= 0.0199809699893635**
		**Std dev= 0.00444304481526729**	**Std dev= 0.00228854730969473**
H10P5	6	0.0215389088059332	0.0226965266484558
		0.0212743565304402	0.0226389399155868
		0.0183953039385395	0.0220064135348744
		0.0225541108528098	0.0249603510982070
		0.0233544438978524	0.0253383918764432
		0.0136272229046643	0.0168696664897599
		**Mean= 0.0201240578217065**	**Mean= 0.0224183815938879**
		**Std dev= 0.00360174947184494**	**Std dev= 0.00303552422636334**
H10P10	6	0.0224919391277188	0.0251018113674805
		0.0208643699870482	0.0296798544897880
		0.0212922066099078	0.0217927961333022
		0.0164994054048487	0.0164353448441155
		0.0147741448352111	0.0146477568859449
		0.0208721604069914	0.0212539195200374
		**Mean= 0.0194657043952877**	**Mean= 0.0214852472067781**
		**Std dev= 0.00307370303551407**	**Std dev= 0.00552507034257499**
H25P5	5	0.0222710365179979	0.0245842753599029
		0.0174708803994585	0.0208463486072463

		0.0174373769495569<br>0.0266831305478903<br>0.0109305738739683<br>**Mean= 0.0189585996577744**<br>**Std dev= 0.00590798697803105**	0.0201035985902860<br>0.0294908162847293<br>0.0176507901290144<br>**Mean= 0.0225351657942358**<br>**Std dev= 0.00461523758747623**
H25P10	5	0.0221765746970105<br>0.0201871269339115<br>0.0152014015933281<br>0.0170512348227609<br>0.0274529325568157<br>**Mean= 0.0204138541207653**<br>**Std dev= 0.00477451788587457**	0.0236787939102669<br>0.0175566690312051<br>0.0165718828944796<br>0.0243735806049649<br>0.0304310405913556<br>**Mean= 0.0225223934064544**<br>**Std dev= 0.00564308980941428**
H25P20	4	0.0246792470152909<br>0.0191693849584367<br>0.0231161676629216<br>0.0307011769611063<br>**Mean= 0.0244164941494389**<br>**Std dev= 0.00478850293047906**	0.0271741777304913<br>0.0323862352390351<br>0.0250586081322511<br>0.0413892180103348<br>**Mean= 0.0315020597780281**<br>**Std dev= 0.00727521583380581**
H50P20	4	0.0222244451914550<br>0.0327859304460666<br>0.0327558092433201<br>0.0343517085617008<br>**Mean= 0.0305294733606356**<br>**Std dev= 0.00558662524439890**	0.0228537439395325<br>0.0562280303184286<br>0.0484340122663414<br>0.0654122697821262<br>**Mean= 0.0482320140766072**<br>**Std dev= 0.0182865602767487**

Agar assay-model fitting SKBR3

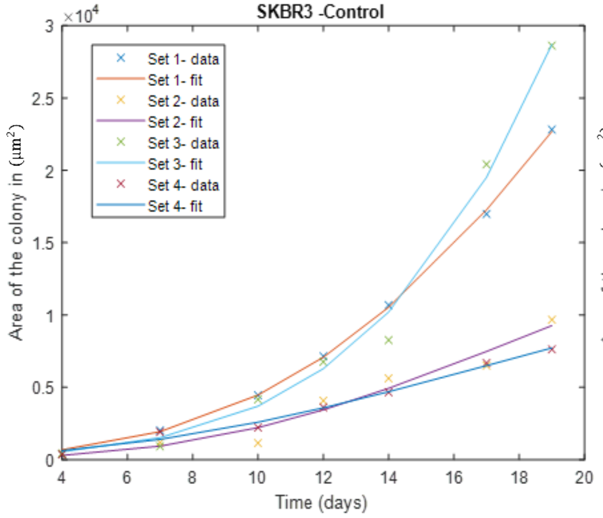

Figure A11: Model fitting curves for SKBR3, 4 sets of control data

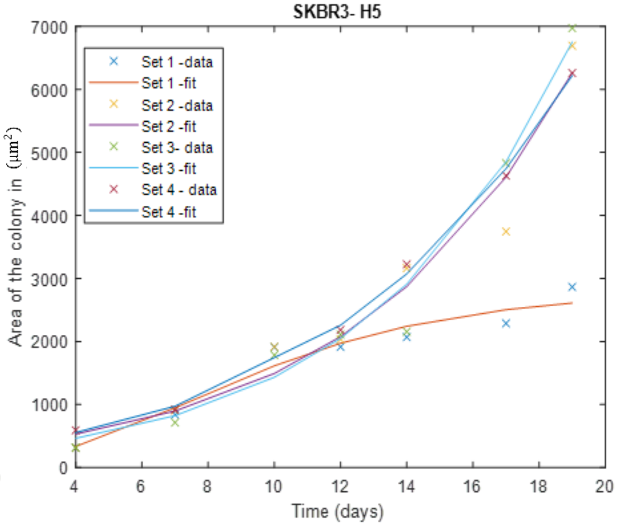

Figure A12: Model fitting curves for SKBR3, 4 sets of H5 data

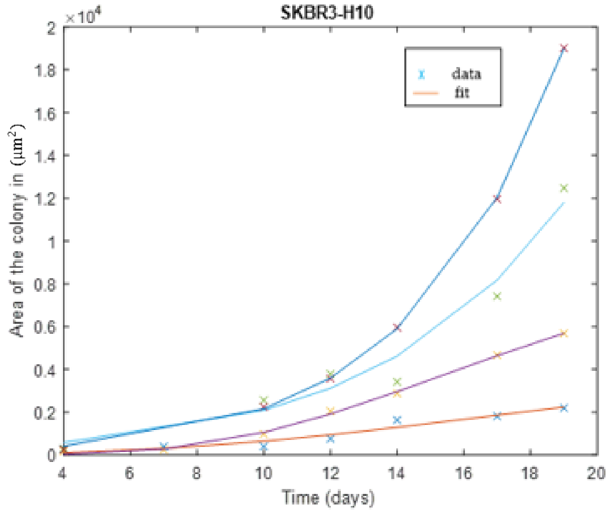

Figure A13: Model fitting curves for SKBR3, 4 sets of H10 data

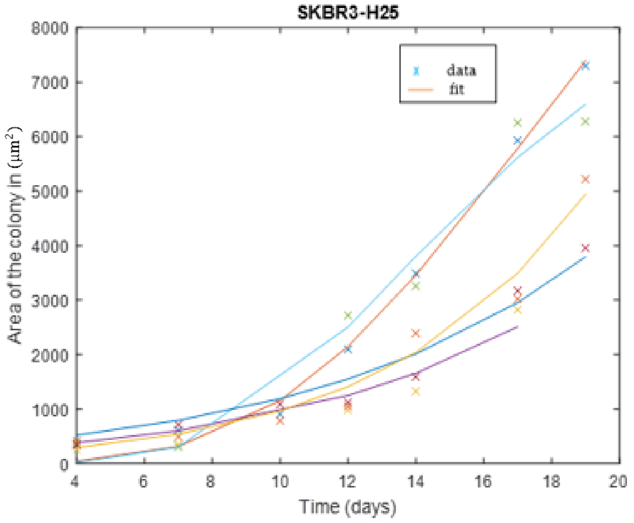

Figure A14: Model fitting curves for SKBR3, 5 sets of H25 data

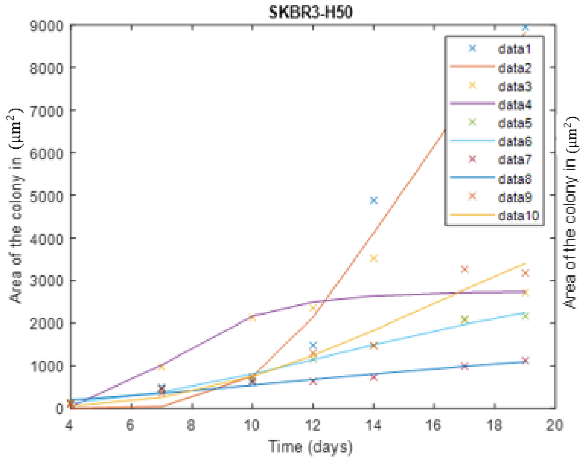

Figure A15: Model fitting curves for SKBR3, 5 sets of H50 data

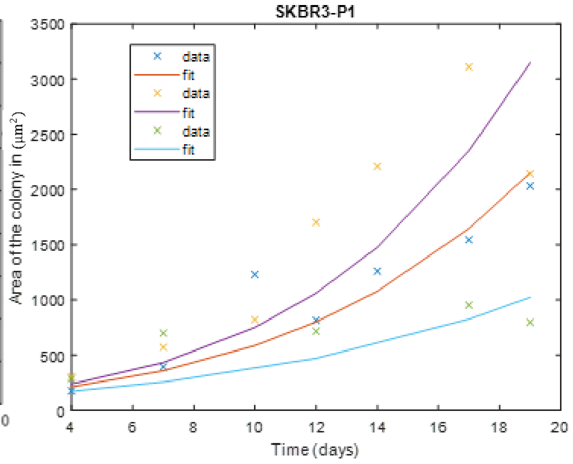

Figure A16: Model fitting curves for SKBR3, 3 sets of P1 data

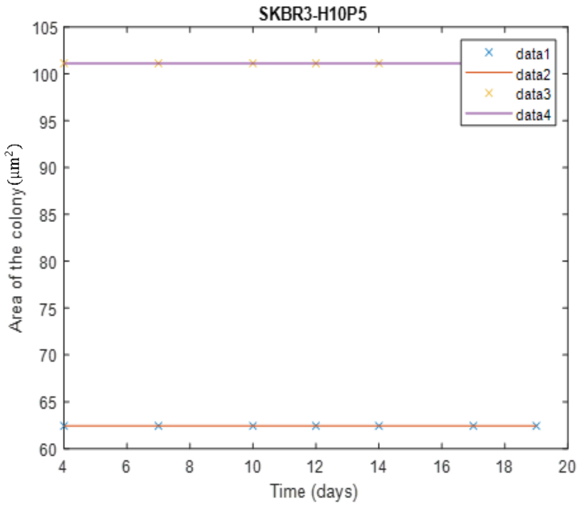

Figure A17: Model fitting curves for SKBR3, 2 sets of H10P5 data

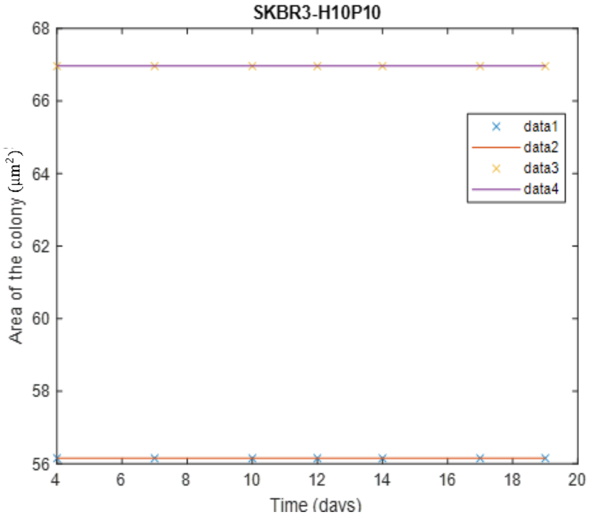

Figure A18: Model fitting curves for SKBR3, 2 sets of H10P10 data

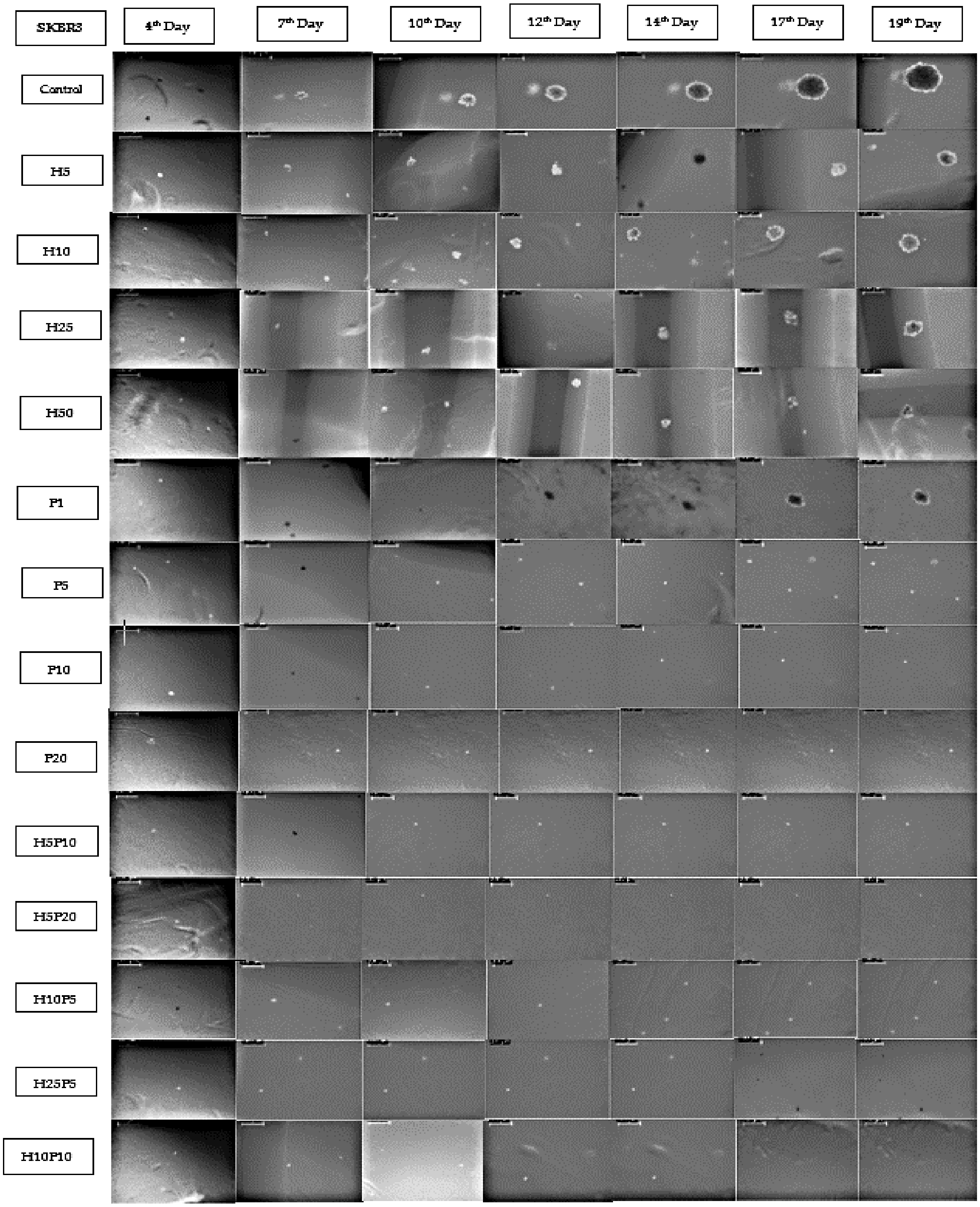

Figure A19: SKBR3 colonies treated with various drug concentrations and combinations. Images are taken using an inverted microscope interfaced to LAS EZ software on 4th, 7th,9th, 10th, 12th, 14th, 17th , and 19th day after seeding. Images are calibrated (scale bar=100μm) using LAZ

EZ software. Images for higher concentrations (H25P10, H25P20, and H50P20) are not shown as the growth inhibition is close to 100%. Some images are repeatedly used in case of missed images due to negligibly small size and size differences (eg. P20, H5P10) due to growth inhibition.

Table AT2: Drug induced growth inhibition of SKBR3 colonies in agar assay. The drug effect parameter a is estimated using model (4) for two cases, case (1) by fixing k=1e6 µm^2, A_0=20 µm^2, and the growth rate of the untreated (control) colony is set as r_c=0.0513, case (2) by fixing k=1e6 µm^2, A_0=100 µm^2, and the growth rate of the untreated (control) colony is set as r_c=0.0423. The overall growth rate of treated colonies is given by $r_{treat} = r_c - a$, and growth inhibition is calculated as % GI=(1-(r_{treat}/r_c))×100.

**Set**	**No. of data set**	**Drug effect (a) days^{-1}, each curve, case 1**	**Drug effect (a) days^{-1}, each curve, case 2**
,Control	4	-0.00536870752089286	-0.00534182078017764
		0.00572253956176294	0.00575974902015577
		-0.00777894256137097	-0.00786043606613477
		0.00731470298270932	0.00742811856596898
		**Mean= -2.76018844478935e-05**	**Mean= -3.59731504691492e-06**
		**Std dev=0.00765036511379465**	**Std dev=0.00771736368716472**
H5	4	0.0170722215642089	0.0172417190891438
		0.0106593009052194	0.0106205582101322
		0.0100450894823899	0.00995358019357872
		0.0105198143755363	0.0105418225649714
		**Mean=0.0120741065818386**	Mean=0.0120894200144565
		**Std dev=0.00334243240430358**	Std dev=0.00344773405593328
H10	4	0.0201841896961720	0.0200712635395184
		0.0112884661209699	0.0112420681627207
		0.00393726000509168	0.00386113680312093
		-0.00145766125830669	-0.00156199388983497
		**Mean=0.00848806364098173**	**Mean=**0.00840311865388124
		**Std dev=0.00938560735476721**	**Std dev=**0.00938328845212817
H25	5	0.00877045988709776	0.00871377614744827
		0.0166310312591332	0.0153890116108234
		0.00937817788621189	0.00943691080739936
		0.0153096775747750	0.0151986782883626
		0.0135197076099392	0.0133596126221231
		**Mean= 0.0127218108434314**	**Mean=**0.0124195978952314
		**Std dev= 0.00351456801970247**	**Std dev=**0.00316440635913231

H50	5	0.00671038720851679<br>0.0164385575357545<br>0.0196616167364397<br>0.0254763986195806<br>0.0164433092625235<br>**Mean= 0.0169460538725630**<br>**Std dev= 0.00680845503588922**	0.00663187528940914<br>0.0168049332327128<br>0.0196364483937999<br>0.0253713951955750<br>0.0163552121565107<br>**Mean=0.0169599728536015**<br>**Std dev= 0.00680027424470143**
P1	3	0.0209724631735786<br>0.0176034855640106<br>0.0269826341988808<br>**Mean= 0.0218528609788233**<br>**Std dev= 0.00475115065014326**	0.0209333939325868<br>0.0177636157259502<br>0.0272924006379431<br>**Mean=0.0219964700988267**<br>**Std dev= 0.00485252858581752**
P5	6	0.0364320943814321<br>0.0343109501757721<br>0.0300952614223026<br>0.0324874588443082<br>0.0340684018816238<br>0.0328351686736422<br>**Mean= 0.0333715558965135**<br>**Std dev= 0.00212312958101281**	0.0361147969461457<br>0.0341755404201526<br>0.0302154412934306<br>0.0324775013419710<br>0.0339511905920245<br>0.0328032465841000<br>**Mean= 0.0332896195296374**<br>**Std dev= 0.00197741376299673**
P10	5	0.0380591523099887<br>0.0355455786054746<br>0.0374111254060514<br>0.0362297511556601<br>0.0385579631939930<br>**Mean= 0.0367607141342336**<br>**Std dev= 0.00125474666162538**	0.0375688489901962<br>0.0353097730235390<br>0.0369936567953401<br>0.0359317884999737<br>0.0380078156763450<br>**Mean= 0.0367623765970788**<br>**Std dev= 0.00112415973954382**
P20	-	-	-
H5P10	5	0.0439436780181482<br>0.0462838784131213<br>0.0448292517138120<br>0.0405269302755651<br>0.0386223081481243<br>**Mean= 0.0428412093137542**<br>**Std dev= 0.00316917713564135**	0.0435403849935141<br>0.0435403849935141<br>0.0435403849935141<br>0.0435403849935141<br>0.0431149896998678<br>**Mean= 0.0434553059347848**<br>**Std dev= 0.000190242558780324**

H5P20	1	0.0453306894108349	0.0435159160240434
H10P5	2	0.0461658274969763<br>0.0422117880171266<br>**Mean= 0.0441888077570514**<br>**Std dev= 0.00279592812928103**	0.0441184475314144<br>0.0411000007567953<br>**Mean=0.0426092241441048**<br>**Std dev= 0.00213436418298385**
H10P10	2	0.0441126819597943<br>0.0440592853966182<br>**Mean= 0.044085983678206**<br>**Std dev= 0.000709841724369373**	0.0447757769522290<br>0.0436828839706297<br>**Mean= 0.0442293304614294**<br>**Std dev= 0.000772792038400040**
H25P5	1	0.0410788690741159	0.0409917241869652
H25P10	1	0.0485553787418886	0.0496389229333788
H25P20	1	0.0519166218427148	0.0510008682314968
